# The Lipopolysaccharide-Induced Metabolome Signature in *Arabidopsis thaliana* Reveals Dynamic Reprogramming of Phytoalexin and Phytoanticipin Pathways

**DOI:** 10.1371/journal.pone.0163572

**Published:** 2016-09-22

**Authors:** Tarryn Finnegan, Paul A. Steenkamp, Lizelle A. Piater, Ian A. Dubery

**Affiliations:** 1 Department of Biochemistry, University of Johannesburg, Auckland Park, 2006, South Africa; 2 CSIR- Biosciences, Natural Products and Agroprocessing Group, Pretoria, 0001, South Africa; Universidad de Malaga, SPAIN

## Abstract

Lipopolysaccharides (LPSs), as MAMP molecules, trigger the activation of signal transduction pathways involved in defence. Currently, plant metabolomics is providing new dimensions into understanding the intracellular adaptive responses to external stimuli. The effect of LPS on the metabolomes of *Arabidopsis thaliana* cells and leaf tissue was investigated over a 24 h period. Cellular metabolites and those secreted into the medium were extracted with methanol and liquid chromatography coupled to mass spectrometry was used for quantitative and qualitative analyses. Multivariate statistical data analyses were used to extract interpretable information from the generated multidimensional LC-MS data. The results show that LPS perception triggered differential changes in the metabolomes of cells and leaves, leading to variation in the biosynthesis of specialised secondary metabolites. Time-dependent changes in metabolite profiles were observed and biomarkers associated with the LPS-induced response were tentatively identified. These include the phytohormones salicylic acid and jasmonic acid, and also the associated methyl esters and sugar conjugates. The induced defensive state resulted in increases in indole—and other glucosinolates, indole derivatives, camalexin as well as cinnamic acid derivatives and other phenylpropanoids. These annotated metabolites indicate dynamic reprogramming of metabolic pathways that are functionally related towards creating an enhanced defensive capacity. The results reveal new insights into the mode of action of LPS as an activator of plant innate immunity, broadens knowledge about the defence metabolite pathways involved in Arabidopsis responses to LPS, and identifies specialised metabolites of functional importance that can be employed to enhance immunity against pathogen infection.

## Introduction

Plants are constantly exposed to a range of environmental stresses including attack by microbial pathogens; however plants have evolved the ability to recognize pathogen-derived molecules as microbe-associated molecular patterns (MAMPs) through non-self recognition [1,2]. Binding to pattern recognition receptors (PRRs) results in the activation of signalling pathways which trigger a complex set of defence mechanisms known as MAMP-triggered immunity (MTI) [1], that includes differential defence-related gene expression and associated protein synthesis. As part of the innate immune system, plants have also developed the ability to enhance resistance to a wide spectrum of potential pathogens in local and distal tissue from the site of infection, in order to launch a more rapid and intense defence response [3,4].

Secondary metabolites play an important role in adaptation and defence during plant-environment interactions. These molecules accumulate as end products of plant metabolomic regulation in response to various abiotic and biotic stresses [5]. As such, metabolomic analyses are increasingly being used for various plant studies including metabolic pathway investigations and plant responses to various stressors [6,7].

*A*. *thaliana*, a member of the Brassicaceae family, produces a number of indolic compounds with camalexin (3-thiazol-2-yl-indole) as the principal phytoalexin [8]. This metabolite plays a role in inhibition of a wide range of bacterial and fungal pathogens as well as abiotic stresses [9]. In turn, the sulfated aldoxine glucosides, or glucosinolates (GSs), have numerous functions in plant adaptation to the environment, particularly in defence against generalist herbivores and microbial pathogens [10,11]. Studies of GSs in plant defence have shown that these antimicrobial glucosides are present at different levels and that the contribution to resistance is dependant on the type of pathogen and infection [12–15]. To gain more insight into the involvement of secondary metabolites in the host responses of *A*. *thaliana* to interactions with herbivores [16], rhizobacteria [17], phyllosphere commensals [18] and phytopathogens [19], metabolomic approaches have been utilised.

Lipopolysaccharides (LPSs) are integral and essential constituents of the outer membrane of Gram-negative bacteria, allowing bacterial growth in hostile environments and promoting attachment to host surfaces [20]. LPSs are recognised as MAMPs [21,22] and previous findings have shown that LPS treatment of Arabidopsis resulted in the induction of a range of defence-related genes, including glutathione *S*-transferases, cytochrome P450s and pathogenesis-related (PR) genes [23,24]. LPSs have been identified as determinants in both induced systemic resistance (ISR) [25] as well as systemic acquired resistance (SAR) [3,26,27], and thus induce an enhanced defensive state in plants [28].

LPSs have complex structures and can potentially contain three different MAMPs; the O-chain, core and lipid A moeities. Consequently, the mechanism by which plants perceive LPS through immunosensors is not fully known. The lipid A moiety may be partly responsible for LPS perception in *A*. *thaliana* [29,30] while results obtained with tobacco suggests additional recognition of the *O*-polysaccharide chain [31]. Recognition of conserved regions by putative LPS PRR(s) results in the activation of calcium influx, initiation of a reactive oxygen species (ROS)—and NO burst and induction of defence responses. These include the hypersensitive response (HR), restricting the pathogen to the site of infection, the production of PR proteins, synthesis of antimicrobial secondary metabolites and cell wall strengthening [23,32,33].

In order to gain more information on the action mechanism of LPSs in the triggering of defence pathways, a metabolomic approach targeted at the secondary defence metabolites was used in this study to analyse LPS-induced changes in defence-related Arabidopsis metabolites. In addition to profiling camalexin, indolic glucosinolates (IGSs) and various indole derivatives, our results provide new insights into the activation of tryptophan-derived defences at an integrated level, and broaden the understanding of plant innate immune responses to LPS. As far as can be ascertained this is the first report on the metabolomic dynamics associated with perception of a specific MAMP in *A*. *thaliana* [34].

## Materials and Methods

### Chemicals and reagents

All reagents used were of analytical grade. Organic solvents, methanol and acetonitrile, were of ultra-pure LC-MS grade (Romil SpS, Cambridge, UK). All equipment was sterilised prior to use, and cell culturing and treatment was carried out under sterile conditions. LPS was purified from *Burkholderia cepacia*, ASP B2D, an environmental strain [26], as previously described [32].

### Plant growth conditions, cell culture and elicitation with lipopolysaccharides

*A*. *thaliana* (ecotype Columbia, Col-0) callus cultures were initiated and grown as previously described [35]. Cells were subcultured onto fresh Murashige and Skoog (MS) agar medium [36] containing 3% sucrose (w/v), 0.8% agar (w/v), 2 mg/mL 2,4-dichlorophenoxyacetic acid, 100 mg/mL myoinositol and B5 vitamins, every 14 days. Plants were grown in germination mix soil (Culterra, Muldersdrif, South Africa) at 23°C, 50% humidity, and 60 μmol m^-2^ sec^-1^ fluorescent illumination in a 12 h light/12 h dark cycle. Six week old plants were used for experiments.

Cells (50 g) were transferred to MS basal salts medium containing LPS (80 μg/mL) in an 1:1.5 m/v ratio, transferred to Falcon tubes and gently mixed to form a suspension. The cell suspensions were divided into four equal aliquots for different time points and kept at 25°C under constant agitation to ensure aerobic conditions. A treatment time study of 8, 12 and 24 h was conducted, and 24 h non-treated cells were used as a negative control. Cell viability–and cell permeability assays were conducted using the triphenyltetrazolium reduction and Evan’s Blue dye uptake methods respectively [26], and did not reveal any detrimental effects of the LPS treatment.

For Arabidopsis leaf elicitation, LPS was dissolved in sterile 10 mM MgSO_4_ to give a final concentration of 80 μg/mL. Arabidopsis plants were treated with the LPS solution by pressure infiltration into the leaves using a blunt-ended syringe. A MgSO_4_-control and non-treated (NT) control were included in the experiments. Following elicitation, four leaves were cut from three different plants (constituting one biological replicate) for the extraction procedure.

Subsequent to optimisation of conditions, each facet of the experimental design was repeated at least three times. Extracts generated from the various experimental treatments were analysed in triplicate (n = 9).

### Metabolite extraction

A methanol-based extraction [7] for polar and semi-polar metabolites was used to isolate metabolites from cells, culture medium and leaf tissue. For rapid quenching of enzyme activity, especially that of myrosinase, hot methanol (55°C) was used to prevent degradation of GSs [37].

Extractions were carried out at 8, 12 and 24 h post-treatment respectively. Cell suspensions (20 mL) were centrifuged at 15 000 x*g* for 15 min, and the supernatants carefully removed and transferred to new 50 mL Falcon tubes. These supernatants, representative of the ‘culture medium’ and containing secreted metabolites, were snap frozen in liquid nitrogen and lyophilised before extraction with methanol. The pellets remaining after centrifugation of the cell suspensions (10 g) were re-suspended in HPLC-grade methanol (1:1.5 m/v), homogenised for 2 min using an Ultraturrax homogenizer (IKA, Staufen, Germany), and centrifuged at 15 000 x*g* for 15 min. The resulting supernatants were collected in new 50 mL Falcon tubes and represented the ‘cell’ fraction. The above extraction protocol was repeated twice and supernatants combined. Extracts were evaporated to 1 mL at 52°C using rotary evaporation under vacuum and the remaining water removed by lyophilization. For both cell- and medium samples, the residues were re-suspended in 2 mL 50% HPLC-grade methanol. The mixtures were vortexed for 30 s and centrifuged at 6000 x*g* for 10 min at 25°C. The supernatants were removed using a syringe and filtered through 0.22 μm nylon syringe filters into glass vials for analyses.

Leaves were cut and 3–5 g transferred to sterile 50 mL Falcon tubes, covered with 80% methanol (1:1 m/v) and homogenised for 3 min. The homogenates were centrifuged for 15 min at 15 000 x*g* and supernatants collected into new Falcon tubes. Methanol was evaporated to 1 mL at 52°C using a rotary evaporator. The residues were then re-suspended in 2 mL 50% HPLC grade methanol and filtered through 0.22 μm syringe filters into glass vials. The extracts were further analysed qualitatively and quantitatively, using various chromatographic techniques and mass spectrometry.

### High-performance thin layer chromatography

HPTLC was performed on 0.2 mm plates pre-coated with silica gel G60 F_254_ (Merck, Darmstadt, Germany). Each sample (20 μL) was spotted 2 cm from the bottom of the plate. The plates were developed using a mobile phase of isopropanol: ethyl acetate: water (7:1:2; v/v/v) [38]. Developed plates were visualised under short (254 nm) and long wave (365 nm) UV light.

### Ultra-high performance liquid chromatography—high definition mass spectrometry

UHPLC-qTOF-MS analyses were performed on a Waters Acquity UHPLC (class ‘Classic’) coupled in tandem to a Waters photodiode array (PDA) detector and a SYNAPT G1 qTOF-mass spectrometer (Waters Corporation, Milford, MA, USA). Chromatographic separation of the metabolites was carried out using a Waters HSS T3 column (150 x 2.1 mm), able to separate both polar and non-polar analytes. A binary solvent mixture of water containing 0.1% formic acid (solvent A) and acetonitrile containing 0.1% formic acid (solvent B) at a flow rate of 0.4 mL/min was used with an injection volume of 5 μL. A gradient protocol with a flow rate of 0.4 mL/min was used. The gradient was set as follows: 5% B over 0.0–1.0 min, 5–90% B over 1.0–10 min, held constant at 90% B over 10.00–12.00 min, and decreasing from 90% to 5% B over 13.00–15.00 min to return to the initial conditions. The PDA detector was set to scan between 200 and 500 nm (1.2 nm resolution) collecting 20 spectra/s. A quality control sample consisting of pooled extracts was used to monitor system stability and Rt reproducibility (technical precision), and was injected every 10 runs.

The qTOF-MS was operated in both positive and negative modes to detect all metabolites of interest. Leucine enkephalin (50 pg/mL) was used as the reference calibrant to obtain typical mass accuracies of between 1 and 3 mDa. A capillary voltage of 2.0 kV with a sampling- and extraction cone voltage of 30.0 V and 4.0 V was used respectively. The scan time was 0.1 s covering a mass range of 100 to 1000 Da. The source temperature was 120°C and the desolvation temperature was set at 400°C. Nitrogen gas was used as the nebulisation gas at a flow rate of 800 L/h. Each sample was analysed in triplicate in a randomised manner.

The MS data was acquired in (MS^E^) mode (a function of the collision cell that alternates between low and high energy states to generate sequential unfragmented and in-source generated fragments) to assist with the annotation and identification of the biomarkers. Here, the MS experiment file was setup to perform unfragmented as well as five fragmenting experiments simultaneously. Ion fragmentation was performed by in-source collision energy ramping (3 eV—30 eV).

Tandem mass spectrometry (MS/MS) analyses were carried out on selected identified biomarkers in order to provide structural information about the compounds of interest, thereby providing an increased level of confidence in the metabolite annotations. The MS method was set up *via* 2 MS functions, outlined in Table A in S1 File. MarkerLynx^TM^ software (Waters Corporation, Manchester, UK) was employed to process and analyse the MS/MS raw data for metabolite identification and structural characterisation. The MarkerLynx^TM^ ‘MassFragment’ tool was used to allocate structures to the fragment ions observed in the LC/MS/MS tandem mass spectra by applying an *in silico* fragmentation algorithm to known precursor structures [39]. The structures of the various fragment ions were then compared to the fragmentation patterns observed in the MS/MS spectra and a putative identification was made.

### Multivariate data analysis (MVDA)

Multivariate statistical analysis was used for the interpretation of LPS-induced metabolomic perturbations and reprogramming seen in the system under study. MVDA methods explain the underlying trends in complex data sets as it allows the analysis of relationships between more than one characteristic at a time.

ESI positive and negative raw data were extracted using MassLynx XS^TM^ software and analysed with the MarkerLynx^TM^ software (Waters Corporation, Manchester, UK). The parameters were set to analyse the 1–13 min retention time (Rt) range of the chromatograms, mass range 100–1000 Da, mass tolerance 0.01 Da, mass window 0.05 Da and a Rt window of 0.20 min.

Following MarkerLynx^TM^ processing, the data matrix was exported into SIMCA-P (Soft independent modelling of class analogy) software, version 12 (Umetrics Corporation, Umea, Sweden), and *Pareto* scaled for principal component analysis (PCA) and orthogonal projection to latent structures discriminant analysis (OPLS-DA) modelling. PCA is an unsupervised mathematical projection-based technique used to reduce the high-dimensionality of complex data sets by producing linear combinations of the original variables called principal components (PCs), ultimately forming lower dimensional data [40,41]. PCA score plots were constructed using mass spectrometric data between Rt 1–13 min with n = 9 datasets.

In turn, OPLS-DA is a supervised, predictive linear regression technique that is an extension of the partial least squares-discriminant analysis (PLS-DA) method where data is modelled according to *a priori* class information (*e*.*g*. Control *versus* Treated) prior to analysis [5]. The OPLS-DA loadings S-plot aided in extracting variables (ions) that are positively correlated to the treatment, and the importance to the model was assessed using the Variable Importance in Projection (VIP) plots (described below).

In order to evaluate the statistical validity for the MVDA models, a number of parameters was considered. The quality of the PCA models was assessed based on the cumulative modelled variation in matrix X, R^2^X(*cum*) and the predictive ability parameter, Q^2^(*cum*), *i*.*e*. the fraction of the total variation of matrix X that can be predicted by the extracted components. For a robust mathematical model with a reliable predictive accuracy, the values of these diagnostic parameters should ideally be above 0.5 or close to 1.0, with the difference between these less than 0.2. For PCA the cumulative modelled variation in X matrix, *R*^*2*^*X* (cum) and the cross-validated predictive ability *Q*^*2*^(cum) values close to 1.0 is an indication that the predictability of the model is reliable [42].

OPLS-DA modelling of control and treated samples was performed using the SIMCA-P software, to separate multivariate relationships into predictive variation (related to LPS treatment) and orthogonal variation (unrelated to LPS treatment). Analysis of variance testing of cross-validated predictive residuals (CV-ANOVA), a diagnostic tool, was used to assess the reliability of the obtained OPLS models with *p*-values < 0.05 indicating a significant model. Metabolites which were affected by the treatment were highlighted as discriminatory ions (signatory biomarkers) by the PCA loading plots and OPLS-DA S-plots. In the case of the latter, only significant metabolites with the correlation [p(corr)] of ≥ 0.6 and covariance of (p1) ≥ 0.5 were chosen for metabolite identification using the *m/z* values to generate elemental composition.

VIP (variable importance in projection) plot analysis, important for elucidation of the X/Y relationship for selection of ions of importance in complex data sets, was performed using SIMCA-P software. The VIP plot is a coefficients plot that represents characteristic X variables associated with the X matrix as well as correlated to the Y response variables. The VIP value summarises the overall contribution of each X-variable summed over all other components and weighted according to the Y variation accounted for by each component [43]. For an ion to be considered relevant, the VIP score should be greater than 1 with increasing VIP scores correlating to increasing significance.

Volcano plots were constructed using MetaboAnalyst 2.0 software (www.metaboanalyst.ca) for further validation of statistically significant compounds found using the OPLS-DA S-plot. Volcano plots are a variant of a scatter plot and used to compare the size of fold change to the statistical significance level. Such univariate analysis, examining each variable separately, aids in extracting potentially important features/ions [44,45]. Volcano plots were constructed using peak intensity tables obtained for both negative—and positive mode data. The parameters used for pre-processing are summarised in Table B in S1 File. The fold change threshold was set at 1.5 with a *p*-value threshold of 0.001.

As described above, for statistical confidence and significance, and to avoid any possible multiple testing problem with regard to the selection of biomarkers, different diagnostic tools and tests (goodness of fit, predictive power estimated using cross-validation, CV-ANOVA, permutation tests, VIP computation, and consideration of magnitude/covariance and reliability/correlation of change) were used to validate and assess the reliability of the computed models, and selection of statistically important variables/biomarkers thereafter.

### Metabolite annotation

Following separation and detection on the UHPLC-qTOF-MS SYNAPT G1, MassLynx^TM^ software was used for analysis of the raw mass spectral data to compute molecular formulae for peaks of interest. The OPLS-DA S-plots were used for prioritising to-be-annotated metabolites that contributed to clustering of samples seen in the PCA plots. The S-plot gives a visual representation of the covariance and correlation from the OPLS-DA scatter plot. From these S-plots, lists of statistically significant and reliable signatory biomarkers were obtained, and putatively annotated according to the Metabolomic Standards Initiative (MSI), level 2 [46].

The molecular formulae of the pseudo-molecular ions ([M-H]^−^ or ([M+H]^+^) representing possible biomarkers were computed and selected based on the criterion that the mass difference between the measured and calculated mass was at/or below 5 mDa. In addition, a number of parameters, including isotopic fit (iFit) and double bond equivalent (DBE) values, were taken into account in order to increase the level of confidence in the molecular formulae obtained. The elemental composition was then searched against online libraries/databases: Dictionary of Natural Products (DNP) (dnp.chemnetbase.com), PubChem (www.pubchem.ncbi.nlm.nih.gov), Chemspider (www.chemspider.com), AraCyc (www.arabidopsis.org/tools/aracyc), PlantCyc (www.plantcyc.org), MetaCyc (www.metaCyc.org), KEGG (www.genome.jp/kegg), Metabolomics workbench (http://www.metabolomicsworkbench.org) and METLIN (metlin.scripps.edu). Moreover, annotation was based on interpretation of mass fragmentation patterns, MS/MS spectra, mass spectral library searches as well as published literature and datasets [47].

Another tool employed in the study for metabolite annotation/putative identification was the PUTMEDID_LC-MS workflow [7,48] that operates on the Taverna workbench (http://www.taverna.org.uk). Putative identification was based on accurate mass data. The data matrix files obtained on the MarkerLynx XS^TM^ software were converted to a version compatible for the PUTMEDID_LC-MS workflow. In workflow 1, a list of pairwise peak correlations were compiled for input into workflow 2. Different ion types of the same metabolite which share similar features (such as Rt and mass differences) were grouped together using basic correlation coefficients such as Pearson or Spearman algorithms (workflow 2). Each accurate *m/z* was then matched to an accurate mass of a neutral compound (reference file) and an elemental composition was calculated (workflow 2) [48]. Lastly, the molecular formulae were searched against accessible online databases/libraries (mentioned above) for identification of metabolites, and a putative identification was made (www.mcisb.org/resources/putmedid.html).

Overall, metabolite assignments should be regarded as annotations or tentative identifications at a metabolite identification (MI)-level 2 [46]. The raw data, together with the study description, have been deposited onto the online data repository, MetaboLights [49], with accession number MTBLS272.

## Results

### Screening for LPS-induced metabolite changes

To target polar and semi-polar compounds which included all classes of GSs and camalexin, a methanol-based extraction procedure was followed. HPTLC analysis was used as an initial screening technique for compounds present in Arabidopsis extracts. Inspection of the chromatograms under long wave (365 nm) UV light showed a time-dependent increase in fluorescent band intensity from control to 24 h-treatment in both the cell and medium samples (Fig A in S2 File). These fluorescent compounds include metabolites with indolic rings such as the IGSs and camalexin [14,15]. Results presented below are from data sets acquired in MS positive mode. Equivalent results for data acquired in MS negative mode are presented as Supplementary files.

### Ultra-high performance liquid chromatography-high definition mass spectrometry

**Fig 1:**
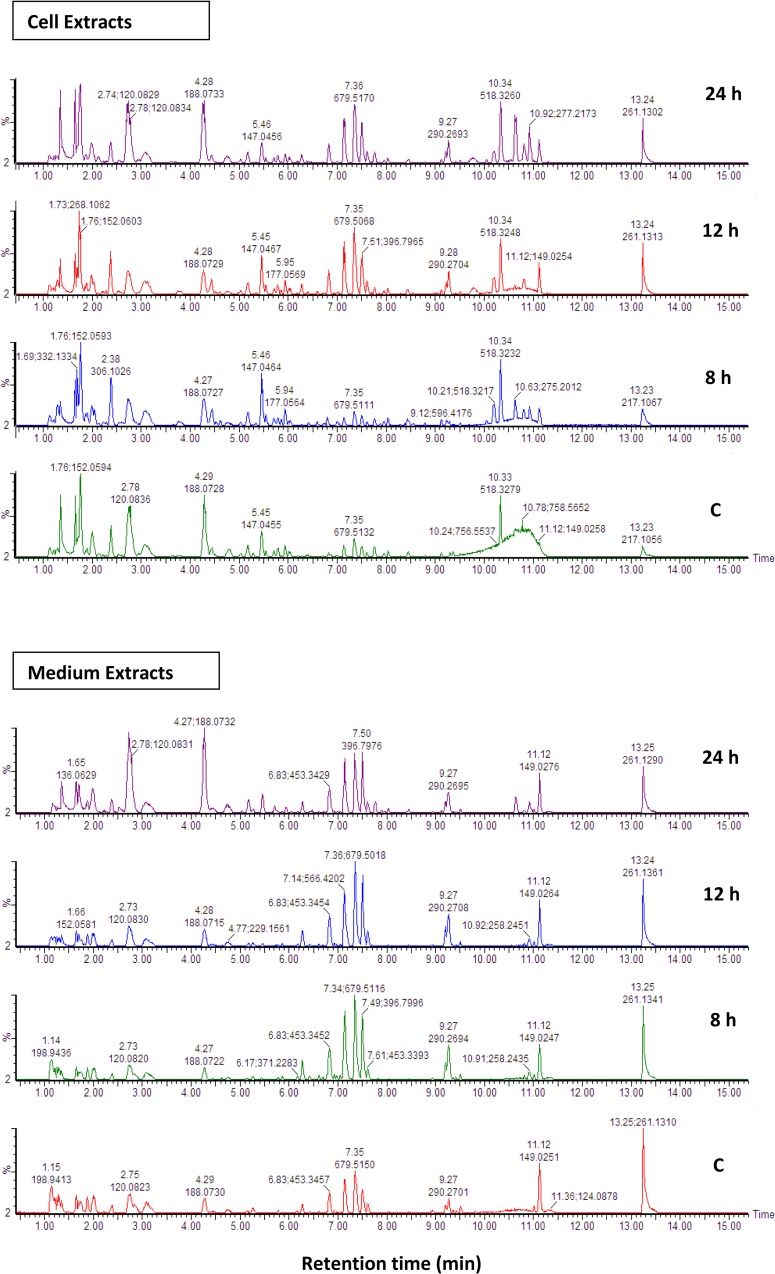
**UHPLC-HDMS (ESI**^**+**^**) BPI chromatograms of LPS-elicited Arabidopsis cell—(A) and growth medium (B) extracts.** Cell suspensions were treated with LPS at a concentration of 80 μg/mL and incubated for different time periods (8, 12 and 24 h) before extraction with methanol. The bottom chromatograms represents the control which was non-treated and incubated for 24 h. The respective Y axes (expressed in %) were linked using the MarkerLynx^TM^ tool for visual comparison.

UHPLC-qTOF-MS was employed to investigate metabolite variations due to LPS treatment during the periods of investigation. MS chromatograms of cell and medium extracts (endo- and exo-metabolomes, respectively) analysed in positive (Fig 1A and 1B) and negative mode (Fig B in S2 File) showed an increase in peak intensity from control to 12 h, followed by a slight decrease in peak intensity at 24 h. Transport of metabolites out of the cell was evident from the positive mode chromatograms for medium extracts (Fig 1B) which showed an increase in peak intensity over time.

The BPI (base peak intensity) MS chromatograms for LPS-treated and control leaves (Fig C in S2 File) showed treatment-related variations in terms of peak intensities and presence/absence of peaks. The MS chromatograms clearly indicate that various compounds in the methanol samples were separated and detected, and that LPS treatment resulted in an altered metabolome in Arabidopsis leaves, as shown by an increase in peak intensities and appearance/absence of peaks. In leaf extracts, two of the dominant peaks showing an increase in intensity in the LPS-treated sample were annotated (based on accurate mass and MS/MS fragmentation patterns and retention times) as glucobrassicin and 4-methoxyglucobrassicin respectively. These IGSs have been reported to be associated with Arabidopsis defence responses, as part of its innate immune system [15] (discussed below).

### Multivariate data analyses of LPS-induced changes in cell, medium and leaf extracts

The obtained UHPLC-MS data were analysed by PCA for unsupervised MVDA modelling, to determine similarities and differences between control and treated samples, as well as between the different time points of the treatment.

**Fig 2:**
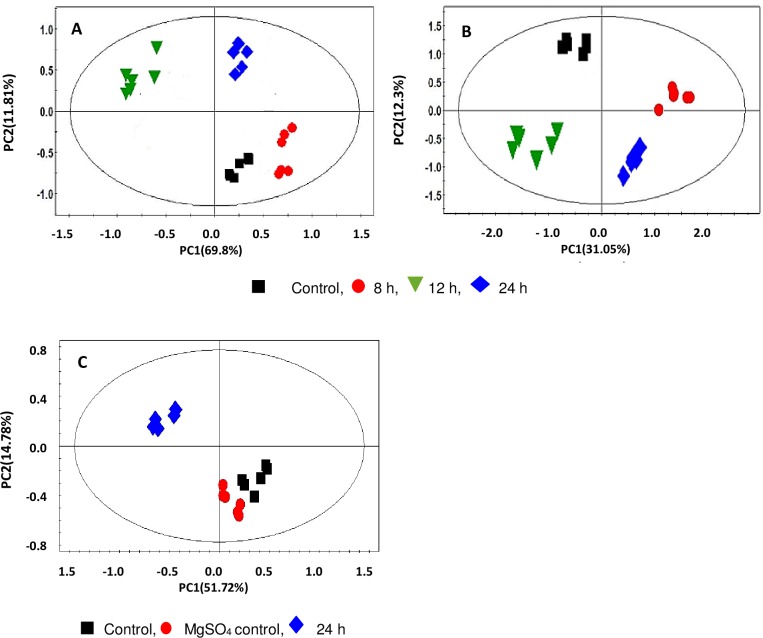
**PCA score plots of metabolite content of extracts from (A) cell, (B) medium and (C) leaf tissue.** Models are based on the UHPLC-qTOF-MS (positive mode) time study of Arabidopsis cell suspensions comparing control versus 8, 12 and 24 h treatments with LPS. Leaf tissue extracts were prepared 24 h post-treatment with LPS and a MgSO_4_ treatment control. The plots show intra- and inter group clustering/separation at different time points. Equivalent plots for the data obtained in negative mode are presented in Fig D in S2 File.

Fig 2A and 2B represents the PCA score scatter plots for data acquired in MS positive mode corresponding to the Rt between 1–13 min. In Fig 2A, cell extract samples (endo-metabolome) clustered into four groups which represent the non-treated (NT) control and treatment time points, indicating chromatographically distinct metabolite profiles and a time-related variation. By comparison, in Fig 2B, culture medium extract samples (exo-metabolome) reflect the secretion of metabolites from the cells and accumulation in the medium.

Fig D in S2 File represents the PCA score plots obtained for leaf extracts analysed in negative mode. The samples clustered into two groups which represents the controls (NT and MgSO_4_) and the LPS-treated samples, indicating a change in the metabolite profile of the leaf tissue. The quality (*R*^*2*^*X* (cum) and *Q*^*2*^(cum), indicating that the predictability of the models is statistically reliable [42]) and statistical validity of the computed PCA models (all ≤ *p* = 0.002) are summarised in Table C in S1 File.

**Fig 3:**
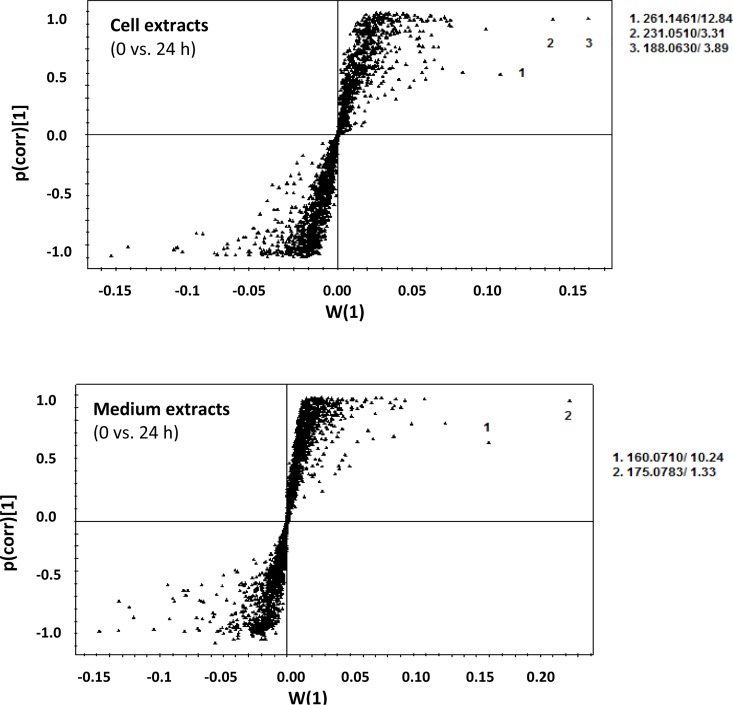
OPLS-DA-based identification of discriminating biomarkers responsible for sample clustering seen in the PCA score plots. Models are based on the UHPLC-qTOF-MS (positive mode) data sets of Arabidopsis cell–and medium extracts comparing control *versus* samples treated with LPS for 24 h. Numbers 1–3 indicate selected variables with *m/z* and Rt indicated. The equivalent plots for the data obtained in negative mode is presented in Fig E in S2 File.

OPLS-DA loadings S-plots (Fig 3 and Fig E in S2 File) were subsequently used to extract variable features (molecular entities characterised by unique *m/z* and Rt values) that were positively or negatively correlated to the treatment. The calculated OPLS-DA models to separate multivariate relationships into predictive variation (related to LPS treatment) and orthogonal variation (unrelated to LPS treatment), were significantly reliable with CV-ANOVA *p*-values of < 0.002 (Table C in S1 File).

**Fig 4:**
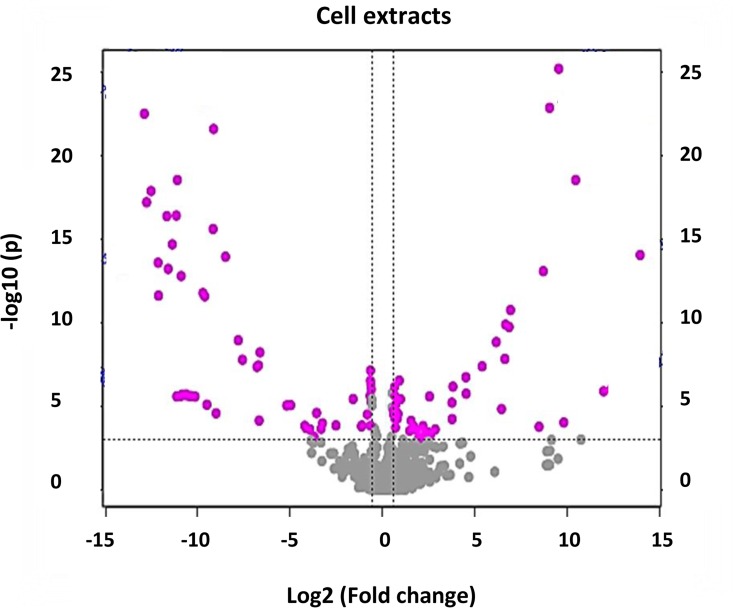
Volcano plot for identification of discriminating biomarkers. Analysis is based on the UHPLC-qTOF-MS (positive mode) time study of Arabidopsis cell extracts comparing control *versus* samples treated with LPS for 24 h. The dashed line shown on the plot indicates where the *p*-value = 0.001, with ions above the line being statistically significant (*p*<0.001). Ions present in the left quadrant of the volcano plot are associated with the NT control and ions in the right quadrant are positively correlated to the treatment. The pink spots represent ions that have a fold change of > 1.5. Ions situated towards the left and right top quadrants represent values of large magnitude fold changes as well as high statistical significance. The equivalent plot for the data obtained in negative mode is presented in Fig E in S2 File.

As a complement to the S-plots, volcano plots were computed to compare the size of fold change to the statistical significance level. Additional mass features of biomarkers could thus be extracted from the generated plots (Fig 4 and Fig E in S2 File). All variable biomarkers (from both the S- and volcano plots) were then annotated as described under Materials and Methods, and are reported in Tables 1 and 2.

**Table 1:** Summary of annotated metabolites, analysed by high definition MS in ESI negative mode, in extracts from LPS-treated *A*. *thaliana* cells and leaf tissues. Metabolites that contributed to the discriminating variability in the altered metabolomes were identified based on OPLS-DA S plots, with VIP-score values >1 indicated. Metabolites annotated from volcano plots are indicated in *italics*. Annotations correspond to the metabolite identification (MI)-level 2 [46].

Metabolites and Categories	*m/z*	Rt (min)	Molecular formula	Adduct	Cell / Medium / Leaf	VIP score
**Defence phytohormones and the precursors/intermediates/conjugates**						
*Salicylic acid*	137.0261	7.05	C_7_H_6_O_3_	[M-H]^-^	C / M	1.98/1.78
4'-Dihydroabscisic acid	265.1431	10.33	C_15_H_22_O_4_	[M-H]^-^	M	1.97
*10-OPDA/12-OPDA/12-oxo-PDA*	291.1966	10.93	C_18_H_28_O_3_	[M-H]^-^	C / M	3.01/1.61
12, 13-Epoxylinolenate	292.2020	10.25	C_18_H_27_O_3_	[M-H]^-^	C / M	2.58/1.51
2-Hydroxy-linoleic acid	295.2263	10.25	C_18_H_32_O_3_	[M-H]^-^	C / M	3.04/1.47
*Salicylic acid β-D-glucoside*	299.0784	4.59	C_13_H_16_O_8_	[M-H]-	C / M	6.97/2.77
Jasmonoyl-L-isoleucine	322.2083	8.88	C_18_H_28_NO_4_	[M-H]^-^	M	2.46
**Branching point between IAA, camalexin and indole glucosinolates**						
*Indole-3-acetaldoxime N-oxide (IAOx)*	235.0451	7.15	C_10_H_10_N_2_O_2_	[M+FA-H]^-^	C	-
**Indole acetic acid and derivatives**						
Indole-3-acetic acid methyl ester	234.0779	5.12	C_11_H_11_NO_2_	[M+FA-H]^-^	C	3.95
Indole-3-acetyl-alanine	267.0752	1.66	C_13_H_13_N_2_O_3_	[M+Na]^-^	M	2.37
Indole-3-acetyl-leucine	309.1207	2.39	C_16_H_19_N_2_O_3_	[M+Na-2H]^-^	C	6.12
*Indole-3-acetyl-beta-D-glucoside*	336.1016	6.16	C_16_H_19_NO_7_	[M-H]^-^	L	-
2-Oxindole-3-acetyl-β-D-glucose (oxIAA-Glc)	352.1119	4.48	C_16_H_19_NO_8_	[M-H]^-^	C	2.86
**Indole carboxylic acid and derivatives**						
Indole-3-carboxylic acid (ICA)	182.0221	3.94	C_9_H_7_NO_2_	[M+Na]^-^	L	2.67
**Desulfoglucosinolate precursors**						
Desulfosinigrin (2-propenyl-desulfoglucosinolate)	279.0777	4.78	C_10_H_17_NO_6_S	[M-H]^-^	C / M	2.43/3.01
Desulfogluconapin (3-butenyl-desulfoglucosinolate)	293.0931	5.35	C_11_H_19_NO_6_S	[M-H]^-^	C / M	6.12/2.88
2-Hydroxy-3-butenyl-desulfoglucosinolate	309.1194	1.61	C_11_H_19_NO_7_S	[M-H]^-^	M	2.55
Desulfoglucoibervirin (3-methylthiopropyl-desulfoglucosinolate)	327.0810	8.49	C_11_H_21_NO_6_S_2_	[M-H]^-^	C / M	2.12/5.87
Desulfoglucotropaeolin (benzyl-desulfoglucosinolate)	329.0933	6.41	C_14_H_19_NO_6_S	[M-H]^-^	C / M	1.92/2.34
Desulfoglucoerucin (4-methylthiobutyl-desulfoglucosinolate)	341.0967	2.12	C_12_H_23_NO_6_S_2_	[M-H]^-^	C / M	2.65/2.27
Desulfoglucoiberin (3-methylsulfinylpropyl-desulfoglucosinolate)	343.1042	2.46	C_11_H_21_NO_7_S_2_	[M-H]^-^	C	1.91
Desulfogluconasturtiin (2-phenylethyl-desulfoglucosinolate)	343.1059	2.46	C_15_H_21_NO_6_S	[M-H]^-^	C	1.91
Desulfoglucoberteroin (5-methylthiopentyl-desulfoglucosinolate)	355.1120	3.90	C_13_H_25_NO_6_S_2_	[M-H]^-^	C	2.13
*Desulfoglucobrassicin (indolylmethyl-desulfoglucosinolate)*	367.0980	6.22	C_16_H_20_N_2_O_6_S	[M-H]^-^	C / L	3.23/-
7-Methylthioheptyl-desulfoglucosinolate	382.1440	1.46	C_15_H_29_NO_6_S_2_	[M-H]^-^	C	3.16
**Indole glucosinolate precursor**						
S-(Indolylmethylthiohydroximoyl)-L-cysteine	292.0741	6.34	C_13_H_15_N_3_O_3_S	[M-H]^-^	M	2.87
**Indole glucosinolates**						
*Glucobrassicin (indol-3-ylmethyl glucosinolate*, *I3G)*	447.0540	4.14	C_16_H_20_N_2_O_9_S_2_	[M-H]^-^	C / M / L	6.12/4.82/16.32
*4-Hydroxyglucobrassicin*	463.0468	3.25	C_16_H_20_N_2_O_10_S_2_	[M-H]^-^	C / M / L	3.44/3.44/4.50
*4-Methoxyglucobrassicin*	477.0648	5.90	C_17_H_22_N_2_O_10_S_2_	[M-H]^-^	C / L	6.91/9.68
*Sulfoglucobrassicin (N-sulfoindol-3-yl)-methyl glucosinolate)*	527.0106	4.10	C_16_H_20_N_2_O_12_S_3_	[M-H]^-^	L	1.71
**Aliphatic glucosinolates: precursors / intermediates**						
*4-Methylthiobutanaldoxime*	134.0561	1.85	C_5_H_11_NOS	[M-H]^-^	C	-
2-Oxo-6-methylthiohexanoate	175.0510	11.17	C_7_H_11_O_3_S	[M-H]^-^	C / M	3.75/3.05
2-Oxo-9-methylthiononanoate	217.0980	4.05	C_10_H_17_O_3_S	[M-H]^-^	C	2.96
9-Methylthiononanaldoxime	292.0963	10.25	C_10_H_21_NOS	[M+FA+Na]^-^	C	2.58
9-Methylthiononylhydroximoyl-L-cysteine	321.1380	6.66	C_13_H_26_N_2_O_3_S_2_	[M-H]^-^	C / L	1.89/3.29
**Aliphatic glucosinolates**						
Gluconapin (butenyl glucosinolate)	354.0322	2.06	C_11_H_19_NO_9_S_2_	[M-H_2_O-H]^-^	M	3.06
*Sinigrin (propenyl glucosinolate)*	358.0281	1.61	C_10_H_17_NO_9_S_2_	[M-H]^-^	M	4.49
Progoitrin (2-hydroxy-3-butenyl glucosinolate)	388.0377	2.37	C_11_H_19_NO_10_S_2_	[M-H]^-^	C / M / L	2.40/2.55/7.81
Glucoerucin (4-methylthiobutyl glucosinolate)	420.0450	8.88	C_12_H_23_NO_9_S_3_	[M-H]^-^	C / L	4.00/8.96
*Glucoberteroin (methylthiopentyl glucosinolate)*	434.0598	5.06	C_13_H_25_NO_9_S_3_	[M-H]^-^	L	2.65
*Glucoraphanin (methylsulfinylbutyl glucosinolate)*	436.0416	1.13	C_12_H_23_NO_10_S_3_	[M-H]^-^	L	8.01
*Glucolesquerellin (6-methylthiohexyl glucosinolate)*	448.0770	5.96	C_14_H_27_NO_9_S_3_	[M-H]^-^	L	7.98
Glucoalyssin (methylsulfinylpentyl glucosinolate)	450.0579	1.25	C_13_H_25_NO_10_S_3_	[M-H]^-^	C / L	4.89/5.75
7-Methylthioheptyl glucosinolate	462.0936	6.62	C_15_H_29_NO_9_S_3_	[M-H]^-^	L	3.83
*Glucohesperin (methylsulfinylhexyl glucosinolate)*	464.0722	1.95	C_14_H_27_NO_10_S_3_	[M-H]^-^	L	2.93
*8-Methylthiooctyl glucosinolate*	476.1095	7.16	C_16_H_31_NO_9_S_3_	[M-H]^-^	L	5.57
*Glucoibarin (methylsulfinylheptyl glucosinolate)*	478.0883	2.06	C_15_H_28_NO_10_S_3_	[M-H]^-^	C / M	4.91/3.62
Glucohirsutin (methylsulfinyloctyl glucosinolate)	492.1034	4.88	C_16_H_31_NO_10_S_3_	[M-H]^-^	L	9.52
*Sinapoylglucoraphenin*	640.0844	2.71	C_23_H_31_NO_14_S_3_	[M-H]^-^	C / M	2.30/4.91
**Aromatic glucosinolates**						
Glucotropaeolin (benzyl glucosinolate)	408.0440	8.55	C_14_H_19_NO_9_S_2_	[M-H]^-^	C	2.75
Gluconasturtiin (2-phenethyl glucosinolate)	422.0590	3.65	C_15_H_21_NO_9_S_2_	[M-H]^-^	L	3.50
**Glucosinolate breakdown products**						
4-Methoxy-3-indolylmethylamine	175.0955	6.56	C_10_H_13_N_2_O	[M-H]^-^	M	3.84
*1-Isothiocyanato-6-(methylthio)hexane*	189.0636	3.12	C_8_H_15_NS_2_	[M-H]^-^	L	-
8-(Methylthio)octylisothiocyanate	216.0888	4.42	C_10_H_19_NS_2_	[M-H]^-^	C	-
**Defence metabolites produced *via* shikimate-phenylpropanoid-flavonoid pathways**						
*Coumaric acid*	163.0412	5.11	C_9_H_8_O_3_	[M-H]^-^	C/M	6.80/1.98
*Vanillic acid (3-methoxysalicylic acid)*	167.0381	3.36	C_8_H_8_O_4_	[M-H]^-^	L	-
Cinnamic acid	193.0514	4.79	C_9_H_8_O_2_	[M+FA-H]^-^	C	2.58
*Cinnamoyl beta-D-glucoside*	310.1012	4.97	C_15_H_18_O_7_	[M-H]^-^	L	-
2,5-Dihydroxybenzoate 2-O-β-D-glucoside	315.0755	2.53	C_13_H_15_O_9_	[M-H]^-^	C / M	3.07/7.73
Coumaric acid-β-D-glucoside	325.0926	3.09	C_15_H_18_O_8_	[M-H]^-^	C / M	2.57/11.87
*Caffeic acid 3-glucoside*	341.0890	2.12	C_15_H_18_O_9_	[M-H]^-^	C / M	2.07/3.89
*Scopolin*	353.2981	6.18	C_16_H_18_O_9_	[M-H]^-^	C / L	2.65/-
*Coniferaldehyde glucoside*	385.1143	5.59	C_16_H_20_O_8_	[M-H]^-^	L	5.0
*Quercetin 3-β-D-glucoside (flavonoid)*	463.0966	6.62	C_21_H_20_O_12_	[M-H]^-^	L	1.78
1,2-bis-*O*-Sinapoyl-beta-D-glucoside	591.1704	7.06	C_28_H_32_O_14_	[M-H]^-^	L	1.74

**Table 2:** Summary of annotated metabolites, analysed by high definition MS in ESI positive mode, in extracts from LPS-treated *A*. *thaliana* cells and leaf tissues. Metabolites that contributed to the discriminating variability in the altered metabolomes were identified based on OPLS-DA S plots, with VIP-score values >1 indicated. Metabolites annotated from volcano plots are indicated in *italics*. Metabolites indicated with an asterix were annotated applying the PUTMED-LCMS workflows [48]. Annotations correspond to the metabolite identification (MI)-level 2 [46].

Metabolites and Categories	*m/z*	Rt (min)	Molecular formula	Adduct	Cell / Medium / Leaf	VIP score
**Defence phytohormones and the precursors / intermediated / conjugates**						
Methylsalicylate	153.0449	1.48	C_8_H_8_O_3_	[M+H]^+^	M	7.49
*Jasmonic acid*	211.1303	12.84	C_12_H_18_O_3_	[M+H]^+^	C / M	4.31/2.65
Linolenic acid	279.2319	10.30	C_18_H_30_O_2_	[M+H]^+^	C	*
*12-OPDA/12-Oxo-PDA*	293.2138	10.23	C_18_H_28_O_3_	[M+H]^+^	M	2.66
17-Hydroxylinolenic acid	295.2200	8.91	C_18_H_30_O_3_	[M+H]^+^	M	2.32
**Branching point between IAA, camalexin and indole glucosinolates**						
Indole-3-acetaldoxime (IAOx)	175.0800	2.74	C_10_H_10_N_2_O	[M+H]^+^	C /M	3.78/12.82
**Indole acetic acid and derivatives**						
*Indole-3-acetic acid (IAA)*	176.1840	3.89	C_10_H_9_NO_2_	[M+H]^+^	C	7.08
2-Oxindole-3-acetic acid (oxIAA)	214.0485	8.30	C_10_H_9_NO_3_	[M+2Na]^2+^	M	4.76
6-Hydroxy-indole-3-acetyl-phenylalanine	339.1255	2.61	C_19_H_17_N_2_O_4_	[M+H]^+^	C	*
7-Hydroxy-2-oxindole-3-acetate glucoside	392.0960	4.03	C_16_H_19_NO_9_	[M+Na]^+^	M	2.23
**Indole carboxylic acid derivatives**						
Indole-3-carboxaldehyde (I3CHO)	146.0600	3.88	C_9_H_7_NO	[M+H]^+^	C / M	*
Indole carbinol (I3C)	148.0763	1.95	C_9_H_9_NO	[M+H]^+^	L	*
**Camalexin biosynthesis**						
*Camalexin*	201.0492	8.78	C_11_H_8_N_2_S	[M + H]^+^	C / M	3.77/2.76
6-Methoxycamalexin (6-methoxy-3-(2-thiazolyl)-1H-indole)	231.0577	3.31	C_12_H_10_N_2_OS	[M + H]^+^	C / M	6.80/2.28
**Desulfoglucosinolate precursors**						
Desulfoglucoerucin (4-methylthiobutyldesulfoglucosinolate)	341.0973	9.65	C_12_H_23_NO_6_S_2_	[M+H]^+^	M	2.38
Desulfoglucolesquerellin (6-(methylthio) hexyldesulfoglucosinolate)	414.0720	8.05	C_14_H_27_NO_9_S_3_	[M+H-2H_2_O]^+^	M	2.28
**Aliphatic glucosinolates: precursors / intermediates**						
*4-Methylthiobutanaldoxime*	134.0561	1.85	C_5_H_11_NOS	[M+H]^+^	C	3.47
2-Oxo-4-methylthiobutanoic acid	149.0220	10.74	C_5_H_7_O_3_S	[M+H]^+^	C / M	4.14/2.16
*6-Methylthiohexanonitrile oxide*	160.0780	6.52	C_7_H_13_NOS	[M+H]^+^	C / M / L	5.39/8.45/4.06
*2-Oxo-5-methylthiopentanoic acid*	163.0427	4.96	C_6_H_10_O_3_S	[M+H]^+^	L	4.05
7-Methylthioheptanonitrile oxide	174.0880	9.99	C_8_H_15_NOS	[M+H]^+^	C	8.64
2-Oxo-6-methylthiohexanoic acid	177.0572	5.31	C_7_H_12_O_3_S	[M+H]^+^	C / M	3.23/2.64
8-Methylthiooctanaldoxime	190.1187	1.98	C_9_H_19_NOS	[M+H]^+^	C / M	4.28/2.32
9-Methylthiononanaldoxime	204.1340	3.8	C_10_H_21_NOS	[M+H]^+^	C / M	3.14/4.21
2-Oxo-8-methylthiooctanoic acid	205.0887	3.97	C_9_H_16_O_3_S	[M+H]^+^	M/ L	2.04/4.05
*2-Oxo-9-methylthiononanoic acid*	219.1028	5.46	C_10_H_17_O_3_S	[M+H]^+^	C	3.25
2-Oxo-10-methylthiodecanoic acid	233.1219	2.58	C_11_H_20_O_3_S	[M+H]^+^	M	1.77
**Aliphatic glucosinolates**						
*Sinigrin (propenyl glucosinolate)*	360.0350	5.66	C_10_H_17_NO_9_S_2_	[M+H]^+^	M / L	1.77/-
*8-Methylthiooctyl glucosinolate*	522.0862	8.63	C_16_H_31_NO_9_S_3_	[M+2Na-H]^+^	C	3.72
Sinapoylglucoraphenin	642.0965	2.18	C_23_H_31_NO_14_S_3_	[M+H]^+^	M	2.28
**Aromatic glucosinolate precursor**						
Phenylacetaldehyde oxime	136.0764	1.33	C_8_H_9_NO	[M+H]^+^	L	1.64
**Aromatic glucosinolate**						
*Gluconasturtiin (2-phenethyl glucosinolate)*	456.1028	6.20	C_15_H_21_NO_9_S_2_	[M+CH_3_OH+H]^+^	L	3.47
**Glucosinolate breakdown products**						
*Phenylacetonitrile*	118.0643	3.87	C_8_H_7_N	[M+H]^+^	M/ L	2.19/-
Raphanusamic acid	162.9720	2.75	C_4_H_4_NO_2_S_2_	[M+H]^+^	C	4.34
Sulforaphane (4-methyl-sulfinylbutyl isothiocyanate)	178.0360	6.55	C_6_H_11_NOS_2_	[M+H]^+^	L	3.08
1-Methoxyindol-3-ylmethyl isothiocyanate	201.0520	2.79	C_11_H_10_N_2_OS	[M+H-H_2_O]^+^	M	1.77
4-Methoxy-3-indolylmethyl isothiocyanate	263.0230	2.45	C_11_H_10_N_2_OS	[M+2Na-H]^+^	M	2.17
**Defence metabolites produced *via the* shikimate-phenylpropanoid-flavonoid pathways**						
5-Hydroxy-coniferaldehyde	195.0590	8.31	C_10_H_10_O_4_	[M+H]^+^	M	11.82
4-Methoxycinnamic acid	201.0520	2.79	C_10_H_10_O_3_	[M+Na]^+^	M	1.77
Quercetin	303.0256	6.23	C_15_H_10_O_7_	[M+H]^+^	C	*
*2*,*5-Dihydroxybenzoate 2-O-β-D-glucoside*	317.0806	2.70	C_13_H_15_O_9_	[M+H]^+^	C / M	3.77/2.42
Quercetin 3-*O-*glucopyranosyl-7-*O*-rhamnopyranoside	611.1530	6.74	C_27_H_30_O_16_	[M+H]^+^	C	*

### Identification of signatory biomarkers and metabolite annotation

Both multivariate OPLD-DA S-plots and univariate volcano plots were constructed to determine statistically significant data points in the cell-, medium–and leaf extracts. The resulting S-plots and volcano plots obtained for extracts analysed for control *vs*. 24 h-treated in positive mode are shown in Fig 3 and Fig E in S2 File respectively. The use of a combination of statistical approaches for extraction of ions related to the LPS treatment ensured a representative picture of the elicited changes to the metabolome under study, as well as clarification of signatory biomarker metabolites contributing to the variation seen in the PCA score scatter plots.

Tables 1 and 2 summarises the data obtained from six separate data sets (Cells, Medium and Leaves, in both negative and positive modes) based on the OPLS-DA and volcano plot analyses. The tables list biomarkers that have a VIP score of > 1 based on OPLS-DA analyses. In addition, biomarkers identified from volcano plot analyses (fold change threshold > 1.5 with a *p*-value threshold of 0.001) are indicated in italics. A total of 64 biomarkers could be annotated from the negative mode data and 42 from the positive mode data.

**Fig 5:**
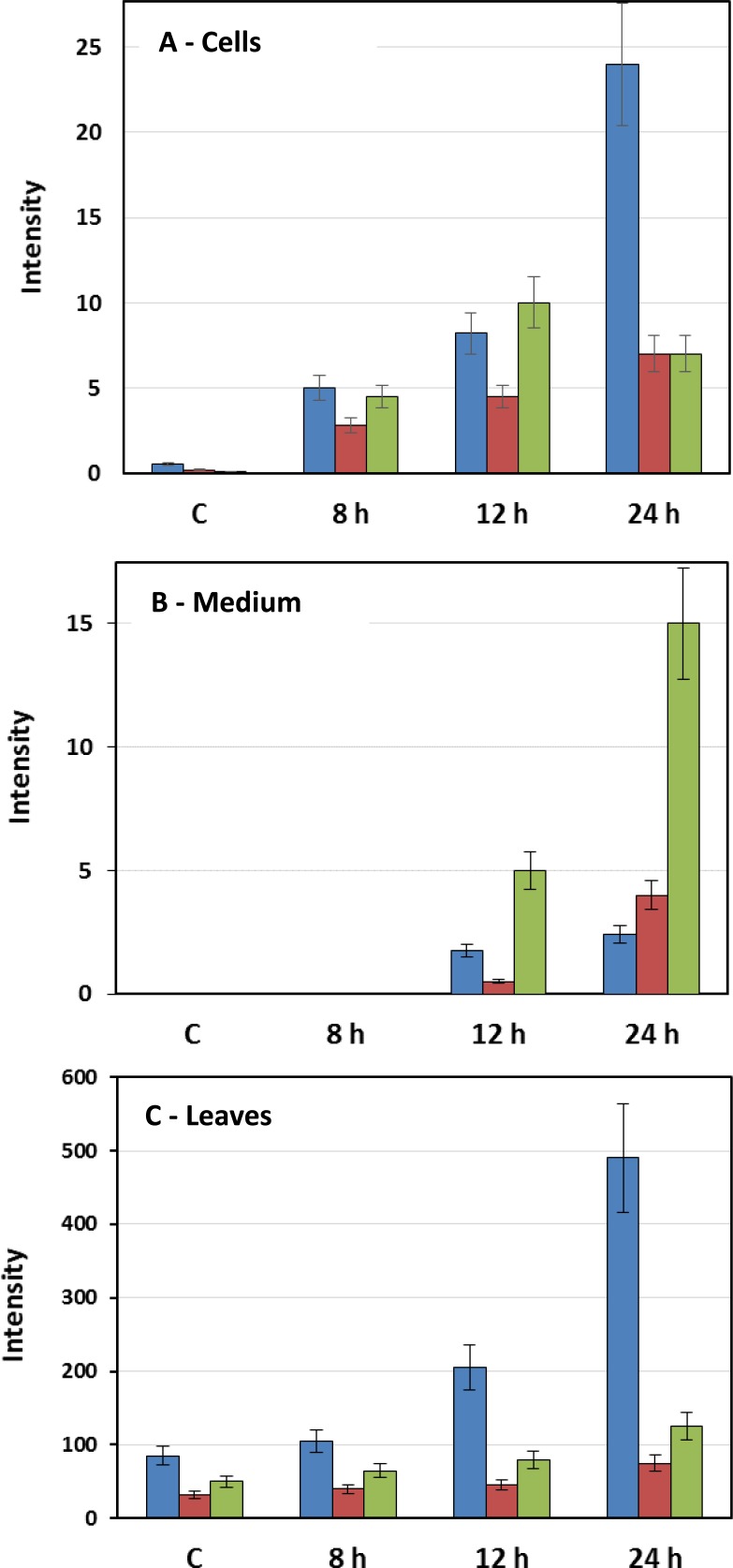
**Graphic presentation of distribution of indole glucosinolates present in (A) cell -, (B) medium—and (C) leaf extracts from Arabidopsis elicited with LPS.** The graphs show the relative concentration, expressed as intensity of integrated ion abundance, for glucobrassicin (blue), 4-hydroxyglucobrassicin (brown) and 4-methoxyglucobrasicin (green) from 8 h to 24 h-treated in comparison to 24 h non-treated controls. Error bars indicate the standard deviation.

In addition to camalexin, GSs from the aliphatic, aromatic and indole classes were found (including precursors, intermediates and degradation products). The tables include the defence phytohormones salicylic acid (SA) and jasmonic acid (JA), as well as metabolites thereof such as methyl salicylate (MeSA), SA glycoside and jasmonoyl-isoleucine (JA-Ile). In addition, metabolites from the phenylpropanoid pathway (cinnamic acid derivatives and glycosides and flavanoid glycosides) were positively annotated. In the case of the IGSs, the distribution of glucobrassicin, 4-hydroxyglucobrassicin and 4-methoxyglucobrassicin present in cell -, medium—and leaf extracts were calculated based on integrated ion abundance data, revealing a time-dependent increase in concentration as presented in Fig 5A–5C. The graphs demonstrate that LPS not only induced the biosynthesis of these 3 IGSs, but patterns within the data are a reflection of time-dependant synthesis, derivitisation (hydroxylation and methylation) and secretion (discussed below).

## Discussion

LPSs as lipoglycan MAMPs induce defence responses at the transcriptomic and proteomic level [23,24,50]. The metabolomics approach followed in this study contributed to clarify metabolite responses of Arabidopsis cells and tissue in countering a perceived microbial attack (as represented by the presence of LPS in the external environment). This contributes to the establishment of a basis to develop targeted approaches for investigating the changes involved in controlling and/or preventing infection by pathogens, and in enhancing host immunity against pathogens as described below. Triggering a combination of SA-responsive SAR and JA-responsive ISR pathways would increase protection against pathogens as well as extend protection against a broader range of pathogens than SAR or ISR alone [51].

High resolution, accurate mass MS is a key analytical platform in plant metabolomics and allows identification of biomarkers with high selectivity and sensitivity. The combination of high mass accuracy (within a few parts per million of the true, calculated, monoisotopic value) and high resolution permits the unambiguous determination of an empirical formula for a mass ion [7,52]. Identified biomarker ions were translated into corresponding metabolite data to understand the occurring metabolic changes. LPS-induced metabolite changes were elucidated; firstly by comparing the BPI MS chromatograms and calculating the molecular formulae for peaks of interest; secondly, by chemometric tools used to extract discriminatory and statistically significant ions; and thirdly by automated approaches, in this case the Taverna PUTMEDID_LC-MS workflow [48].

From all the PCA score plots, the LPS-induced metabolic changes in both cell cultures and leaves were evident. The S-plots and volcano plots indicated *m/z* ions that exhibit high magnitude, high correlation and statistical significance. This approach was used for comparison of data from control extracts *versus* extracts obtained from the 8, 12 and 24 h treatments. In addition, MS/MS analyses were performed to fragment specific sample ions to assist in metabolite annotation.

### Defence-associated changes in the metabolome

SA and the conjugate, SA β-D-glucoside (SAG), were among the list of annotated metabolites, and are known to accumulate in areas surrounding infection sites associated with the HR response as well as distal areas [53]. SA is a key signalling molecule in plant defence and associated with SAR responses, the up-regulation of PR proteins and increased accumulation of phenylpropanoid compounds [3] which all contribute to limiting pathogen infection and by creating an antimicrobial environment. In Arabidopsis, camalexin synthesis is also under the control of SA-signalling [8]. In addition, JA and JA-ileu were also annotated to be positively correlated to the LPS treatment. In contrast to the SA and SAR link, JA is associated with ISR responses [25] and contributes to a signalling cascade involved in production of GSs [54]. These findings corroborate our previous observations that LPS trigger both SA and JA signalling events as indicated by activated gene expression of the *PR-1* (pathogenesis-related 1) and *PDF1*.*2* (plant defensin) marker genes [23]. Although SA and JA hormone regulated pathways generally interact antagonistically [4], a recent study [55] provided evidence that these pathways can work synergistically as part of a defence response against pathogens.

GS composition of *A*. *thaliana* varies considerably between populations and environmental factors play a role [56,57]. Significant differences also occur between tissues and organs [58]. A complex genetic network controls GS biosynthesis and accumulation [54,59]. The upregulation of GS production in response to biotic stressors are mediated in part by JA, SA and ethylene, where different defence pathways activate subsets of biosynthetic enzymes, leading to the accumulation of specific GSs [60]. To modulate the IGS biosynthetic pathway, feedback inhibition by the accumulation of IGS and IGS hydrolytic products occur [15].

Based on transcriptome and co-expression data of stress- and hormone-responsive MYB transcription factors, a reciprocal negative feedback control mechanism between the IGS and aliphatic GS pathways in Arabidopsis leaves was proposed [59]. No confirmatory conclusions about such an inverse relationship could be made based on our metabolome data which identified both IGSs and aliphatic GSs as LPS-responsive signatory biomarkers (Tables 1 and 2).

A number of indolic-, aliphatic- and aromatic GSs were found in cell-, medium- and leaf extracts (Tables 1, 2 and Table D in S1 File). In addition to the intact GSs, desulfoglucosinolates (dsGSs) belonging to all three classes were annotated as biomarkers. Transfer of the sulfate moiety to the dsGS is the final biosynthesis step and these findings indicate that GS biosynthesis was ongoing at the sampled time points.

In addition to IGSs, camalexin was amongst the metabolites present in both cell- and medium extracts. This may be indicative of passive diffusion but recent data reported the active translocation of IGSs out of the cell for defence purposes when needed [61]. It is now realised that a different activation principle and end-products are engaged in GS defence responses to microbial pathogens as compared to insect herbivory. The suggested function of IGSs in Arabidopsis MAMP-triggered immunity is independent from cellular destruction, and involves a distinctive pathway for IGS conversion involving PENETRATION2 (PEN2, an atypcial myrosinase) activity and secretion of bioactive products to the cell periphery / apoplast [14,15,61]. From a functional apoplastic immunity perspective, this is very important since secretion of defence-related metabolites to sites of early pathogen infection (apoplast, cell wall, phylloplane, etc.), is essential to act as a deterrent.

In addition to the IGSs, GSs belonging to the aromatic and aliphatic classes were also identified as being positively correlated to the LPS treatment through the MVDA. Although regarded as phytoanticipins, increased production of aliphatic and aromatic GSs as well as altered profiles may occur in response to abiotic or biotic stresses [62] as supported by our results.

Other non-GS metabolite annotations (Tables 1 and 2) included compounds from the shikimate-phenylpropanoid-flavonoid pathways which is explained by SA as a response marker. SA-responsive cinnamic acid derivatives such as *p*-coumaric acid have been reported to act as precursors for a broad range of phenylpropanoid derivatives with antimicrobial activity and as precursors (*e*.*g*. coniferaldehyde) in lignin synthesis [63].

A comparison of results from cells and leaves indicated that fewer bio-markers were identified in leaf tissue compared to the undifferentiated cells in suspension, possibly indicating more stringent metabolic control in the differentiated tissues at organ level complexity. Overall, the results obtained with cell cultures and leaf tissue indicate broadly similar responses to LPS perception by dynamic changes in the metabolome, especially activation of the IGS pathway as a defence mechanism.

### The indole metabolite profile of LPS-activated defence responses

Tryptophan metabolism, leading to camalexin and IGS biosynthesis, plays an important role in various aspects of pre-and post-invasive non-host resistance in Arabidopsis [64]. The Trp-derived specialised metabolites are inter-related through a common precursor namely indole-3-acetaldoxime, (IAOx) [65]. IAOx represents a major branch point between the synthesis of camalexin, IGSs and indole acetic acid (IAA) [10,14,66].

We have previously reported the upregulated expression of the IAOx-producing *CYP79B2* in response to LPS where, under the same experimental conditions as used in this study, camalexin levels increased 40 fold over a 24 h period [33]. As part of the metabolomic study, camalexin was again identified as an LPS responsive signatory biomarker. In addition to camalexin, the annotation of the biomarker (*m/z* = 231.0577) as 6-methoxy-camalexin is of special interest since Arabidopsis is not generally known to accumulate this metabolite. However, a related observation was of a sugar conjugate of 6-hydroxy-camalexin, that occurs at low levels in Arabidopsis leaves treated with silver nitrate and *Phytophthora infestans* [67].

The annotated IGSs that were found to be upregulated included glucobrassicin, 4-hydroxyglucobrassicin, 4-methoxyglucobrassicin and sulphoglucobrassicin from both cell, medium and leaf extracts. Glucobrassicin and 4-hydroxyglucobrassicin showed a time-dependent response in cells treated with LPS (Fig 5A). Concentration levels increased from 8 to 24 h treatment in both cell- and medium extracts. In the cell extracts, 4-methoxyglucobrassicin was found to accumulate significantly 12 h post-treatment and decreased slightly thereafter. This could be the result of transport thereof out of the cell (as shown by the significant increase in concentration of 4-methoxyglucobrassicin in the medium sample (Fig 5B) 12 h post-treatment). A similar pattern emerged from the results obtained with extracts from leaf tissue, where glucobrassicin was the dominant IGS to accumulate (Fig 5C).

IGSs are normally found at relatively low concentrations in the vegetative stage of rosette leaves [58], but pathogen inoculation or MAMP treatment redirects IGS biosynthesis to 4-substituted IGSs [14,15,68]. Hydroxylation reactions at the GS indole ring are catalysed by members of the subfamily CYP81F of cytochrome P450s, and the resulting hydroxy intermediates serve as substrates for subsequent methoxylation. In the case of 4-hydroxy-IGS, this reaction has been proposed to be carried out by a SA-responsive IGS *O*-methyltransferase (IGMT) [60,69]. Products of IGS metabolism are required for pathogen resistance in Arabidopsis [61] and have been proposed to control entry of certain fungal and oomycete pathogens into the epidermal cells. In addition, these compounds can affect callose deposition and programmed cell death [70]. Breakdown products of the IGSs include indolyl-3-ylmethylisothiocyanate, indol-3-ylmethylamine (here detected and annotated as the 4-methoxy derivatives) and raphanusamic acid. Furthermore, indolyl-3-ylmethylisothiocyanate reacts rapidly to form the antimicrobial indole-3-carbinol (I3C) and/or indole-3-acetonitrile (IAN), the camalexin precursor [61].

2-Oxindole-3-acetic acid (oxIAA) and the oxidised conjugate, 7-hydroxy-2-oxindole-3-acetate glucoside (oxIAA-Glc), are biomarkers of IAA metabolism. The former is a major catabolite of IAA and generated in response to increased levels thereof. This metabolite was proposed as an important element in the regulation of auxin homeostasis and response mechanisms [71], and the presence in Arabidopsis cells in response to LPS exposure is an indication of the increased flux in the metabolic pathways originating from tryptophan.

Indole-3-carboxaldehyde (I3CHO) and indole-3-carboxylic acid (I3COOH) have been regarded as breakdown products of IAA. However, I3COOH was found to accumulate in the wall-bound cellular fraction, especially in response to incompatible interactions [72]. Furthermore, the concentrations of I3CHO and I3COOH and derivatives thereof have been reported to increase upon pathogen infection, indicating a link to defence [14,72]. I3CHO is converted to I3COOH by an inducible ARABIDOPSIS ALDEHYDE OXIDASE 1 (AA01) that is coexpressed with camalexin biosynthesis genes [73]. Recent reports indicate that I3COOH is specifically primed by beta-aminobutyric acid upon *Plectosphaerella cucumerina* infection [74]. When applied as a priming agent, it induces resistance in Arabidopsis against *P*. *cucumerina* [75]. Recently, a new branch of indole metabolism leading to 4-hydroxyindole-3-carbonyl nitrile (4-OH-ICN) was discovered in Arabidopsis [76]. Pre-treatment with 4-OH-ICN also conferred greater resistance to *Pseudomonas syringae* infection, supporting a direct mechanism of action in inducible plant defence. This adds support for the concept of the biosynthesis of an inducible and complex blend of phytoalexins consisting of induced and defence-related indolic compounds [68].

**Fig 6:**
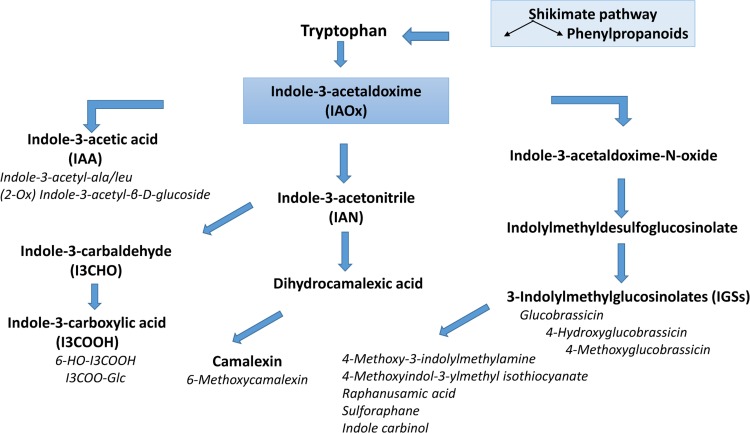
The indolic metabolite footprint of LPS-triggered signalling in Arabidopsis. In response to LPS perception, enhanced activity of CYP79B2/B3 (Beets et al. 2012) converts Trp and lead to the accumulation of indole-3-acetaldoxime (IAOx), the common precursor of indole glucosinolates (IGSs), indole phytoalexins (camalexin), indole-3-carboxaldehyde (I3CHO), indole-3-carboxylic acid (I3COOH) and indole acetic acid (IAA). Associated derivatives and conjugates are indicated in italics. The antimicrobial roles of the annotated metabolites (*e*.*g*. phytoalexins, phytoanticipins and priming agents) and the metabolic interrelationships are discussed in the main text.

The identification of indole carboxylic acid derivatives, together with camalexin and the IGSs, is an indication of an interconnected metabolic network or grid [10]. Synergistic actions between the metabolites would strengthen the combined antimicrobial effect, contributing to increased protection against pathogens with different mechanisms of attack and pathogenesis. The various branches of the Trp-derived defence metabolites are thus integrated into the framework of antimicrobial innate immune responses that are initiated following the perception of LPS by putative PRR(s) (Fig 6).

## Conclusion

In the current metabolomic study, the combination of liquid chromatography coupled to mass spectrometry (UHPLC-MS) techniques provided a broad picture of the effect of LPS perception on the defence metabolome of Arabidopsis. This illustrates the unprecedented power of metabolomics as an effective and unbiased approach in studying cellular biochemistry of an induced defence response at a global level.

Perception of LPS by Arabidopsis cells and leaves resulted in metabolic adaptations as reflected by time-dependent and treatment-related profile variations as detected by UHPLC-MS. MVDA statistical and metabolomic tools provided a visual image of the similarities and differences (sample clustering), and thus allow identifation of patterns in the data based on changes in peak intensities and appearance/ absence of peaks. These metabolic changes not only include variation in metabolite levels, but also production of new metabolites. Results obtained reveal new aspects of the Arabidopsis response to LPS and indicate that cellular (in either an undifferentiated or differentiated state) perception of LPSs leads to significant alterations of the metabolomes, thus triggering differential responses which include the production of specialised metabolites utilised in a defence context.

The presence of indolic compounds (IAA, I3CHO, I3COOH and associated derivatives), camalexin, four IGSs (glucobrassicin, 4-methoxyglucobrassicin, 4-hydroxyglucobrassicin and sulfoglucobrasscin), aliphatic- and aromatic GSs and other metabolites from the shikimate-phenylpropanoid-flavonoid pathways as biomarkers, indicate the breadth of the observed defence-associated reprogramming in the Arabidopsis metabolome. This study therefore provides further insight into the potential of Arabidopsis to evoke metabolism of Trp-derived metabolites as part of its innate immunity in response to MAMPs as exemplified by LPSs. The overall defence responses of plants are complex processes which change depending on environmental conditions and the species involved. Understanding the relationship between the Arabidopsis metabolome and an inducible resistance phenotype would require further analysis of the accumulation of the indole-derivatives, camalexin and GSs, and defence-associated breakdown / utilisation at the organ, tissue, cellular and subcellular level.

The annotation of SA and JA represents another notable dimension of our study since these phytohormones are general biomarkers of biotic stress. The annotation thereof as signatory biomarkers indicates that both of these signalling defence pathways are activated in Arabidopsis in response to perception of the molecular patterns within LPS, thus tracing the footprint of LPS on indole-containing metabolites back to the mechanism as an inducer of enhanced resistance. This is an important finding that advances our understanding of the potential roles of LPSs as inducers of a stress- and defence responses in SAR and ISR.

## Supporting Information

S1 File **Table A,** Settings used for MS/MS analyses on the Waters UHPLC-qTOF Synapt G1 qTOF-MS system. **Table B,** Data pre-processing steps for the construction of volcano plots. **Table C,** Quality and reliability of computed PCA and OPLS-DA models. **Table D,** Table of systematic- and common names for glucosinolates identified in *A*. *thaliana* responding to LPS elicitation.
(PDF)

S2 File **Fig A, Changes in the fluorescent metabolite profiles indicative of the *A*. *thaliana* cellular response to LPS elicitation.** Representative HPTLC chromatograms of extracts prepared from LPS-treated *A*. *thaliana* cells incubated over various time periods (C, 8 h, 12 h, 24 h) in growth medium. The non-treated control was incubated for 24 h. Fluorescent compounds were visualised under UV light (360 nm). Relative fluoresence profiles reflect the dynamic changes in the indole-containing metabolites (summarised in Fig 6). **Fig B, UHPLC-qTOF-MS (negative mode) base peak intensity (BPI) chromatograms of LPS-elicited Arabidopsis (A) cell and (B) culture medium extracts.** Cell suspensions were treated with LPS at a concentration of 80 μg/mL and incubated for different time periods (8, 12 and 24 h) before extraction with methanol. The bottom chromatogram represents the control which was non-treated and incubated for 24 h. The respective Y axes (expressed in %) were linked using the MarkerLynx^TM^ tool for visual comparison. **Fig C, UHPLC-qTOF-MS BPI chromatograms of the Arabidopsis leaf extracts in (A) negative and (B) positive MS modes.** Leaves were elicited with LPS for 24 h and extracted as described. Controls include a NT control (C1) and a 8 mM MgSO_4_ control (C2) which were incubated for 24 h. Dominant peaks 1 and 2 were annotated as glucobrassicin and 4-methoxyglucobrassicin respectively. The respective Y axes (expressed in %) were linked using the MarkerLynx^TM^ tool for visual comparison. Retention times are staggered along the X-axis to ease comparison of the chromatograms. **Fig D, PCA score plots of cell (A), medium (B) and leaf (C) extracts**. Models are based on UHPLC-qTOF-MS data (negative mode) of Arabidopsis cells and leaves were treated with LPS as described. The plots show intra- and inter group clustering/separation at different time points, indicating ongoing changes in the respective metabolomes: Control, 8 h, 12 h and 24 h for cell- and medium extracts (A and B), and control and 24 h for leaf extracts with an additional MgSO_4_ treatment control as indicated (C). **Fig E, Identification of discriminating biomarkers based on the UHPLC-qTOF-MS (negative mode) time study of Arabidopsis cell extracts, comparing control versus samples treated with LPS for 24 h.** (A) OPLS-DA-derived S-plot for identification of discriminating variables responsible for sample clustering seen in the PCA score plots. (B) Volcano plot. The dashed line shown on the plot indicates where the p-value = 0.001 with ions above the line being statistically significant (p<0.001). Ions present in the left quadrant of the volcano plot are associated with the NT control, and ions in the right quadrant are positively correlated to the treatment. The pink spots represent ions that have a fold change of > 1.5. Ions situated towards the left and right top quadrants represent values of large magnitude fold changes as well as high statistical significance.
(PDF)
