# Supplementary material for: The Lipopolysaccharide-Induced Metabolome Signature in Arabidopsis thaliana Reveals Dynamic Reprogramming of Phytoalexin and Phytoanticipin Pathways
Source: PLoS One. 2016 Sep 22;11(9):e0163572. doi: 10.1371/journal.pone.0163572 (PMC5033345; doi:10.1371/journal.pone.0163572)
Supplement: S1 File — Table A, Settings used for MS/MS analyses on the Waters UHPLC-qTOF Synapt G1 qTOF-MS system. Table B, Data pre-processing steps for the construction of volcano plots. Table C, Quality and reliability of computed PCA and OPLS-DA models. Table D, Table of systematic- and common names for glucosinolates identified in A. thaliana responding to LPS elicitation. (PDF) [file pone.0163572.s001.pdf]

The lipopolysaccharide-induced metabolome signature in *Arabidopsis thaliana* reveals dynamic reprogramming of phytoalexin and phytoanticipin pathways.

Tarryn Finnegan[1], Paul A Steenkamp[1,2], Lizelle A Piater[1] and Ian A Dubery[1]*

[1]Department of Biochemistry, University of Johannesburg, Auckland Park, 2006 South Africa and [2]CSIR- Biosciences, Pretoria 0001, South Africa.

S1 File - Index of Supporting Information

A Table. Settings used for MS/MS analyses on the Waters UHPLC-qTOF Synapt G1 qTOF-MS system.

B Table. Data pre-processing steps for construction of volcano plots.

C Table.
(A) The quality of the computed PCA models described as the cumulative modelled variation in X matrix, R^2X (cum), and the cross validated predictive ability values, Q^2(cum). If the values for R^2X (cum) and Q^2(cum) are close to 1.0, it indicates that the predictability of the model is statistically reliable (Mavel *et al.*, 2013). Models were generated using SIMCA-P ver. 12 software.
(**B**) OPLS-DA modeling of Control and LPS-treated samples was performed and analysis of variance testing of cross-validated predictive residuals (CV-ANOVA), a diagnostic tool, was used to assess the reliability of the obtained OPLS models (SIMCA-P-ver. 12 software).

D Table. Known glucosinolates identified in *A. thaliana* with systematic- and common names.

A Table.
Settings used for MS/MS analyses on the Synapt G1 UHPLC-qTOF-MS system.

**Function 1**	**Function 2**
Try to keep the compound(s) from fragmenting by running low energies	Try to fragment the compound(s) without totally fragmenting the pseudomolecular ion $[M+H]^+$ or $[M-H]^-$
Capillary voltage = 2000 V	Capillary voltage = 2000 V
Cone voltage = 30 V	Cone voltage = 30 V
Trap Energy voltage = 3 V	Trap collision Energy Ramp voltage = 10-20 V or 20-30 V or 30-40 V (MS methods with different second MS functions)
Transfer Energy voltage = 1 V	Transfer Energy voltage = 1 V
Mass scan range = 100-1000 Da	Mass scan range = 100-1000 Da
Set mass = Mass of compound to be detected [M+H]+ or [M-H]- and specified to two decimals	Set mass = Mass of compound to be detected $[M+H]^+$ or $[M-H]^-$ and specified to two decimals
Lockmass specified to 4 decimal places	Lockmass specified to 4 decimal places
Scan time = 0.1 min.<br>Data collected in Centroid mode	Scan time = 0.1 min.<br>Data collected in Centroid mode

B Table.
Data pre-processing steps for construction of volcano plots*.

**Pre-processing step**	
Data filtering	By standard deviation option
Data normalization	By median
Data transformation	Log_{10} transformation
Data scaling	*Pareto* scaling
Fold change threshold	1.5
p-value threshold	0.001

* Volcano plots were constructed by plotting the negative log_{10} of the *p*-value on the Y-axis. Consequently, datapoints with low *p*-values (highly significant) appear toward the top of the plot. The X-axis represents the log_2 of the fold change between the LPS-treated and control, so that changes in both directions appear equidistant from the center. Two regions of interest are then apparent in the plot: those points that are found toward the top of the plot that are far to either the left- or the right-hand side. These represent signatory biomarkers that display large magnitude fold changes (being left- or right- of center) as well as high statistical significance (being toward the top).

C Table.

(A) The quality of the computed PCA models described as the cumulative modelled variation in X matrix, R^2X (cum), and the cross validated predictive ability values, Q^2(cum). If the values for R^2X (cum) and Q^2(cum) are close to 1.0, it indicates that the predictability of the model is statistically reliable (Mavel *et al*., 2013). Models were generated using SIMCA-P ver. 12 software.

PCA models	R^2X (cum)	Q^2 (cum)	Variability from PC1 and PC2
**ESI^- MS data**			
Fig. S2D-A - Cells	0.784	0.858	57.02%
Fig. S2D-B - Medium	0.826	0.763	57.2%
Fig. S2D-C - Leaves	0.723	0.889	66.09%
**ESI^+ MS data**			
Fig. 2-A - Cells	0.762	0.821	81.69%
Fig. 2-B - Medium	0.868	0.820	53.35%
Fig. 2-C - Leaves	0.857	0.802	66.5%

(B) OPLS-DA modelling of Control and LPS treated samples was performed and analysis of variance testing of cross-validated predictive residuals (CV-ANOVA), a diagnostic tool, was used to assess the reliabilitry of the obtained OPLS models (SIMCA-P-ver.12 software).

OPLS-DA models	***P* values**	
	**ESI^- MS data**	**ESI^+ MS data**
**Cells**		
C vs 8h	< 0.001	0.002
C vs 12 h	< 0.001	< 0.001
C vs 24 h	< 0.001	< 0.001
**Medium**		
C vs 8 h	< 0.001	< 0.001
C vs 12 h	< 0.001	0.001
C vs 24 h	< 0.001	< 0.001
**Leaves**		
C vs 24 h	< 0.001	< 0.001

D Table.

Table of systematic- and common names for glucosinolates identified in *A. thaliana* (Kliebenstein *et al.*, 2001; Petersen *et al.*, 2002; Brown *et al.*, 2003; Arany *et al.*, 2008).

Glucosinolate type*	Systematic name	Common name	Found**
	**Aliphatic glucosinolates**		
Methylsulfinylalkyl side chains	Methylsulfinylpropyl glucosinolate	Glucoiberin	○
	Methylsulfinylbutyl glucosinolate	Glucoraphanin	●
	Methylsulfinylpentyl glucosinolate	Glucoalyssin	●
	Methylsulfinylhexyl glucosinolate	Glucohesperin	●
	Methylsulfinylheptyl glucosinolate	Glucoibarin	●
	Methylsulfinyloctyl glucosinolate	Glucohirsutin	●
Methylthioalkyl side chains	Methylthiopropyl glucosinolate		
	Methylthiobutyl glucosinolate	Glucoerucin	○,●
	Methylthiopentyl glucosinolate	Glucoberteroin	○,●
	Methylthiohexyl glucosinolate	Glucolesquerellin	○
	Methylthioheptyl glucosinolate		○,●
	Methylthiooctyl glucosinolate		●
Alkenyl side chains	Propenyl glucosinolate	Sinigrin	○,●
	Butenyl glucosinolate	Gluconapin	○,●
	Pentenyl glucosinolate	Glucobrassicanapin	
Hydroxyalkyl side chains	Hydroxypropyl glucosinolate		
	Hydroxybutyl glucosinolate		
Other side chains	(2R)-2-Hydroxy-3-butenylglucosinolate	Progoitrin	○,●
	(2S)-2-Hydroxy-3-butenylglucosinolate	Epiprogoitrin	
	(2S)-2-Benzoyloxy-3-butenylglucosinolate		
	**Indolic glucosinolates**		
Derived from tryptophan	Indol-3-ylmethyl glucosinolate	Glucobrassicin	○,●
	4-Methoxyindol-3-yl methylglucosinolate	4-Methoxy glucobrassicin	●
	1-Methoxyindol-3-yl methylglucosinolate	Neoglucobrassicin	
	4-Hydroxyindol-3-yl methylglucosinolate	4-Hydroxy glucobrassicin	●
	(N-sulfoindol-3-yl)-methyl glucosinolate)	Sulfoglucobrassicin	●
	**Aromatic glucosinolates**		
Derived from phenylalanine	Benzyl glucosinolate	Glucotropaeolin	○,●
	2-Phenylethyl glucosinolate	Gluconasturtiin	○,●
	2-Hydroxy-2-phenyletyl glucosinolate	Glucobarbarin	
	3-Benzoyloxypropyl glucosinolate	Glucomalcomiin	
	4-Benzoyloxybutyl glucosinolate		
Derived from tyrosine	4-Hydroxy benzyl glucosinolate	Glucosinalbin	

*Glucosinolates are diverse in their origins, side chain modification, degradation and final biological functions (Grubb and Abel, 2006). They comprise short- and long-chain aliphatic glucosinolates (Ile, Leu, Val, Ala and Met), indolic glucosinolates (Trp) and aromatic glucosinolates (Tyr and Phe). Met, Trp and Phe have been confirmed as precursors in *A. thaliana*.

** Matched annotation as a LPS-responsive signatory biomarker, either as desulfoglucosinolate precursor (○) or as the glucosinolate (●).
