# Supplementary material for: The Lipopolysaccharide-Induced Metabolome Signature in Arabidopsis thaliana Reveals Dynamic Reprogramming of Phytoalexin and Phytoanticipin Pathways
Source: PLoS One. 2016 Sep 22;11(9):e0163572. doi: 10.1371/journal.pone.0163572 (PMC5033345; doi:10.1371/journal.pone.0163572)
Supplement: S2 File — Fig A, Changes in the fluorescent metabolite profiles indicative of the A. thaliana cellular response to LPS elicitation. Representative HPTLC chromatograms of extracts prepared from LPS-treated A. thaliana cells incubated over various time periods (C, 8 h, 12 h, 24 h) in growth medium. The non-treated control was incubated for 24 h. Fluorescent compounds were visualised under UV light (360 nm). Relative fluoresence profiles reflect the dynamic changes in the indole-containing metabolites (summarised in Fig 6). Fig B, UHPLC-qTOF-MS (negative mode) base peak intensity (BPI) chromatograms of LPS-elicited Arabidopsis (A) cell and (B) culture medium extracts. Cell suspensions were treated with LPS at a concentration of 80 μg/mL and incubated for different time periods (8, 12 and 24 h) before extraction with methanol. The bottom chromatogram represents the control which was non-treated and incubated for 24 h. The respective Y axes (expressed in %) were linked using the MarkerLynxTM tool for visual comparison. Fig C, UHPLC-qTOF-MS BPI chromatograms of the Arabidopsis leaf extracts in (A) negative and (B) positive MS modes. Leaves were elicited with LPS for 24 h and extracted as described. Controls include a NT control (C1) and a 8 mM MgSO4 control (C2) which were incubated for 24 h. Dominant peaks 1 and 2 were annotated as glucobrassicin and 4-methoxyglucobrassicin respectively. The respective Y axes (expressed in %) were linked using the MarkerLynxTM tool for visual comparison. Retention times are staggered along the X-axis to ease comparison of the chromatograms. Fig D, PCA score plots of cell (A), medium (B) and leaf (C) extracts. Models are based on UHPLC-qTOF-MS data (negative mode) of Arabidopsis cells and leaves were treated with LPS as described. The plots show intra- and inter group clustering/separation at different time points, indicating ongoing changes in the respective metabolomes: Control, 8 h, 12 h and 24 h for cell- and medium extracts (A and B), and cont [file pone.0163572.s002.pdf]

The lipopolysaccharide-induced metabolome signature in *Arabidopsis thaliana* reveals dynamic reprogramming of phytoalexin and phytoanticipin pathways.

Tarryn Finnegan[1], Paul A Steenkamp[1,2], Lizelle A Piater[1] and Ian A Dubery[1]*

[1]Department of Biochemistry, University of Johannesburg, Auckland Park, 2006 South Africa and
[2]CSIR- Biosciences, Pretoria 0001, South Africa.

S2 File - Index of Supporting Information

A Fig. Changes in the fluorescent metabolite profiles indicative of the *A. thaliana* cellular response to LPS elicitation. Representative HPTLC chromatograms of extracts prepared from LPS-treated *A. thaliana* cells incubated over various time periods (C, 8 h, 12 h, 24 h) in growth medium. The non-treated control was incubated for 24 h. Fluorescent compounds were visualised under UV light (360 nm). Relative fluoresence profiles reflect the dynamic changes in the indole-containing metabolites (summarised in Fig 6).

B Fig. UHPLC-qTOF-MS (negative mode) base peak intensity (BPI) chromatograms of LPS-elicted Arabidopsis (A) cell and (B) culture medium extracts. Cell suspensions were treated with LPS at a concentration of 80 µg/mL and incubated for different time periods (8, 12 and 24 h) before extraction with methanol. The bottom chromatogram represents the control which was non-treated and incubated for 24 h. The respective Y axes (expressed in %) were linked using the MarkerLynx^{TM} tool for visual comparison.

C Fig. UHPLC-qTOF-MS BPI chromatograms of the Arabidopsis leaf extracts in (A) negative and (B) positive MS modes. Leaves were elicited with LPS for 24 h and extracted as described. Controls include a NT control (C1) and a 8 mM $MgSO_4$ control (C2) which were incubated for 24 h. Dominant peaks 1, 2 and 3 were annotated as glucobrassicin, 4-hydroxyglucobrassicin and 4-methoxyglucobrassicin respectively. The respective Y axes (expressed in %) were linked using the MarkerLynxTM tool for visual comparison. Retention times are staggered along the X-axis to ease comparison of the chromatograms.

D Fig. PCA score plots of cell (A), medium (B) and leaf (C) extracts. Models are based on UHPLC-qTOF-MS data (negative mode) of Arabidopsis cells and leaves were treated with LPS as described. The plots show intra- and inter group clustering/separation at different time points, indicating ongoing changes in the respective metabolomes: Control, 8 h, 12 h and 24 h for cell- and medium extracts (A and B) and Control and 24 h for leaf extracts with an additional $MgSO_4$ treatment control as indicated (C).

E Fig. Identification of discriminating biomarkers based on the UHPLC-qTOF-MS (negative mode) time study of Arabidopsis cell extracts, comparing control versus samples treated with LPS for 24 h. (A) OPLS-DA derived S-plot for identification of discriminating variables responsible for sample clustering seen in the PCA score plots. (B) Volcano plot. The dashed line shown on the plot shows where the p-value = 0.001 with ions above the line being statistically significant ($p<0.001$). Ions present in the left quadrant of the volcano plot are associated with the NT control and ions in the right quadrant are positively correlated to the treatment. The pink spots represent ions that have a fold change of > 1.5. Ions situated towards the left and right top quadrants represent values of large magnitude fold changes as well as high statistical significance.

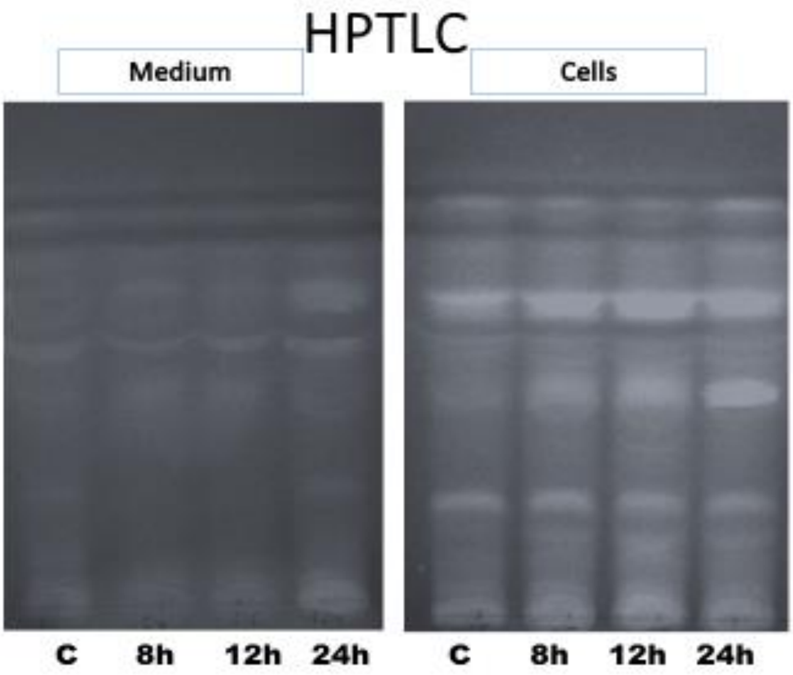

A Fig. Dynamic changes in the fluorescent metabolite profiles indicative of the *A. thaliana* cellular response to LPS elicitation. Representative HPTLC chromatograms of extracts prepared from LPS-treated *A. thaliana* cells incubated over various time periods (C, 8 h, 12 h, 24 h) in growth medium. The non-treated control was incubated for 24 h. The plates show fluorescent compounds visualised under UV light (360 nm) to detect time-dependent variation in band intensity. Relative fluoresence profiles reflect the dynamic changes in the indole-containing metabolites (summarised in Fig 6).

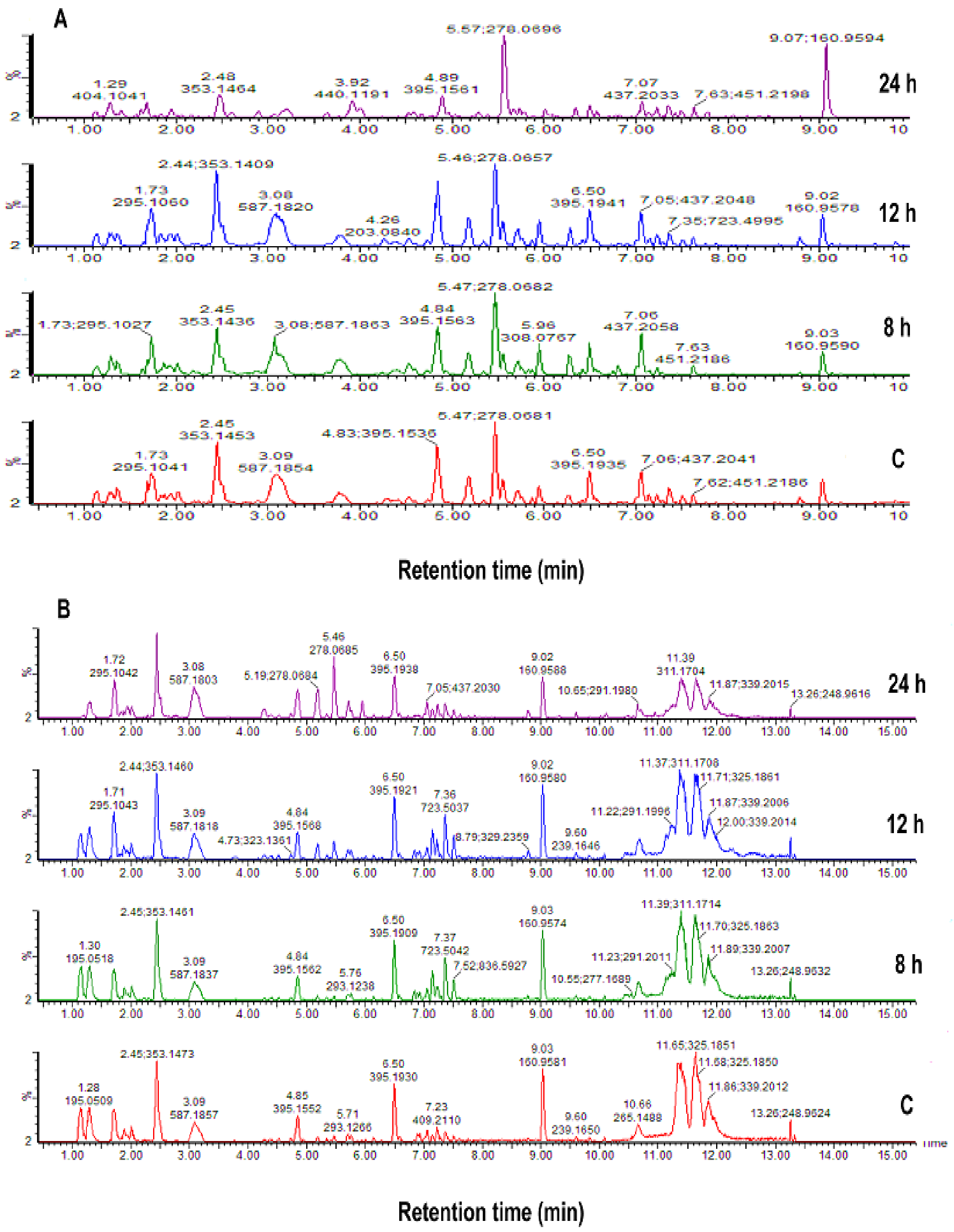

B Fig. **UHPLC-qTOF-MS (negative mode) base peak intensity (BPI) chromatograms of LPS-elicted Arabidopsis (A) cell and (B) culture medium extracts.** Cell suspensions were treated with LPS at a concentration of 80 µg/mL and incubated for different time periods (8, 12 and 24 h) before extraction with methanol. The bottom chromatogram represents the control which was non-treated and incubated for 24 h. The respective Y axes (expressed in %) were linked using the MarkerLynx™ tool for visual comparison.

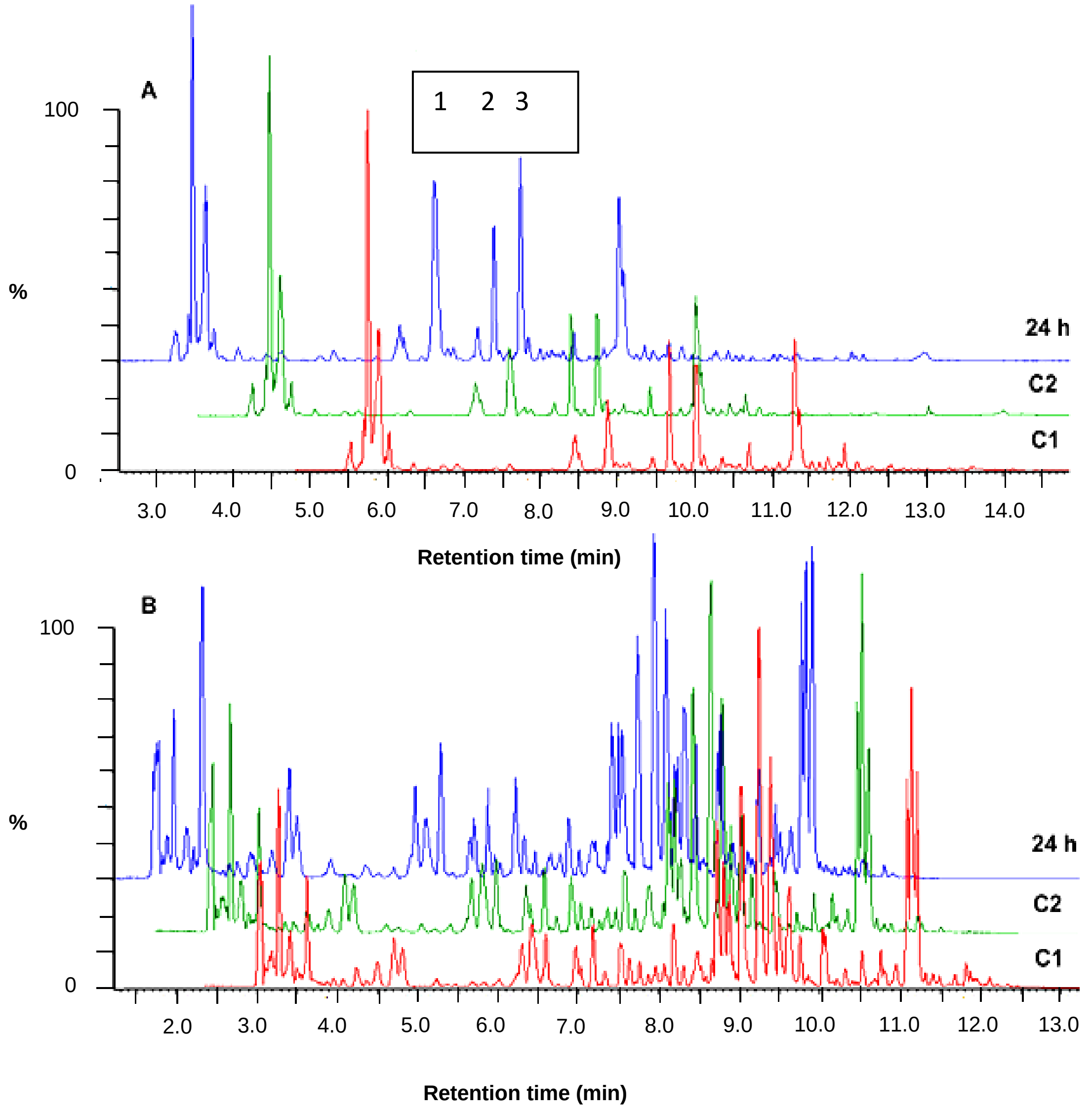

<u>C Fig.</u> UHPLC-qTOF-MS BPI chromatograms of the Arabidopsis leaf extracts in (A) negative and (B) positive MS modes. Leaves were elicited with LPS for 24 h and extracted as described. Controls include a NT control (C1) and a 8 mM $MgSO_4$ control (C2) which were incubated for 24 h. Dominant peaks 1, 2 and 3 were annotated as glucobrassicin 4-hydroxyglucobrassicin and 4-methoxyglucobrassicin respectively. The respective Y axes (expressed in %) were linked using the MarkerLynxTM tool for visual comparison. Retention times are staggered along the X-axis to ease comparison of the chromatograms.

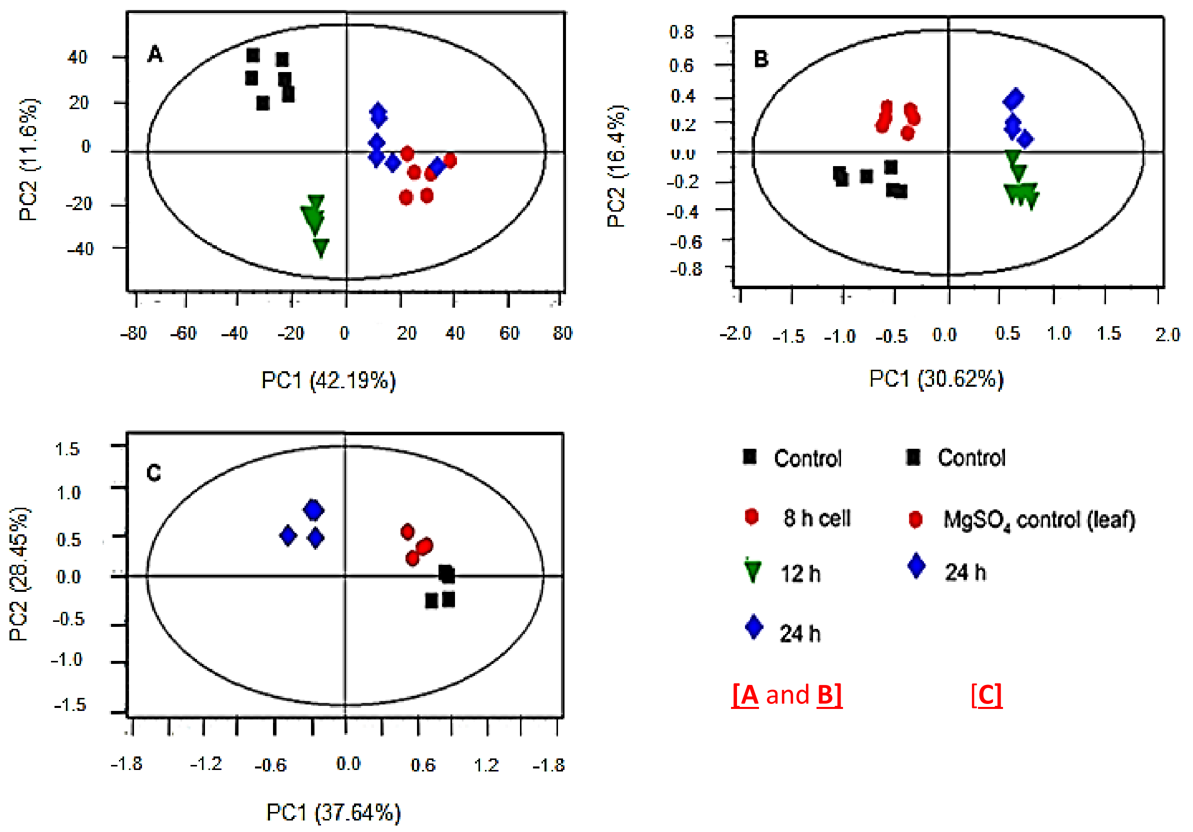

D Fig. PCA score plots of cell (A), medium (B) and leaf (C) extracts. Models are based on UHPLC-qTOF-MS data (negative mode) of Arabidopsis cells and leaves were treated with LPS as described. The plots show intra- and inter group clustering/separation at different time points, indicating ongoing changes in the respective metabolomes: Control, 8 h, 12 h and 24 h for cell- and medium extracts (A and B) and Control and 24 h for leaf extracts with an additional $MgSO_4$ treatment control as indicated (C).

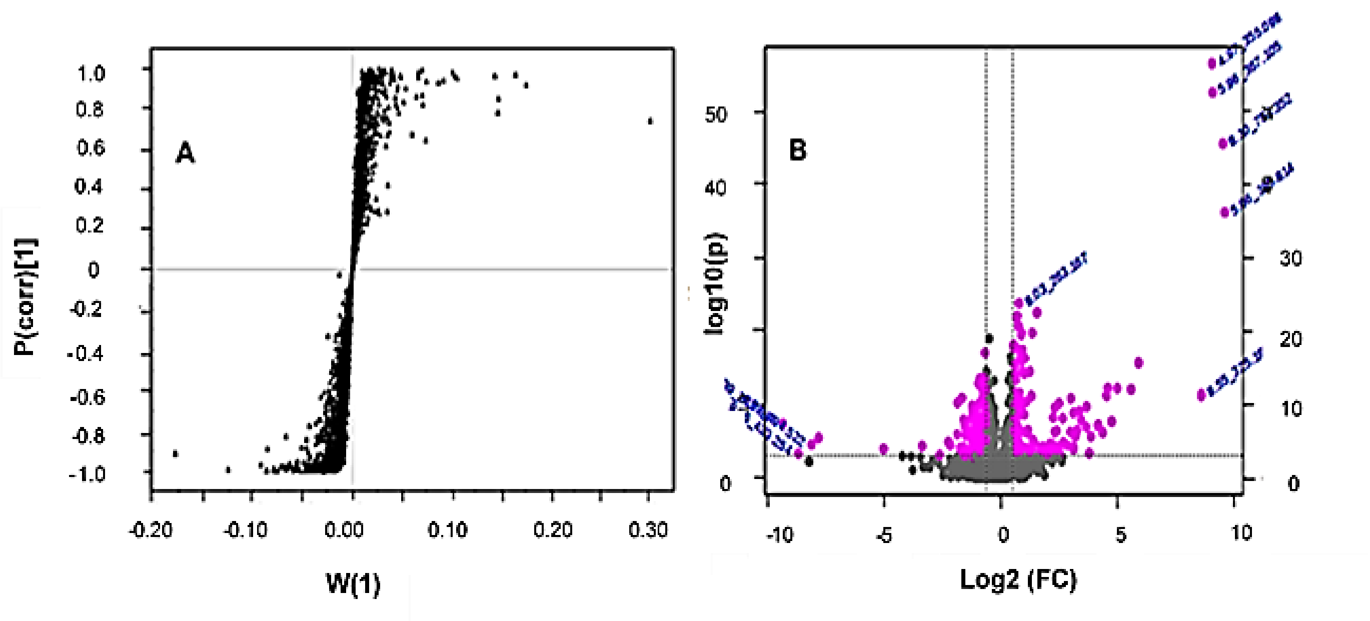

<u>E Fig.</u> Identification of discriminating biomarkers based on the UHPLC-qTOF-MS (negative mode) time study of Arabidopsis cell extracts comparing control versus samples treated with LPS for 24 h.
(A) OPLS-DA derived S-plot for identification of discriminating variables responsible for sample clustering seen in the PCA score plots.
(B) Volcano plot. The dashed line shown on the plot shows where the p-value = 0.001 with ions above the line being statistically significant ($p<0.001$). Ions present in the left quadrant of the volcano plot are associated with the NT control and ions in the right quadrant are positively correlated to the treatment. The pink spots represent ions that have a fold change of > 1.5. Ions situated towards the left and right top quadrants represent values of large magnitude fold changes as well as high statistical significance.
